# Supplementary figures and images for: Increased brain size of the dwarf Channel Island fox (Urocyon littoralis) challenges “Island Syndrome” and suggests little evidence of domestication
Source: PLoS One. 2025 Aug 20;20(8):e0328893. doi: 10.1371/journal.pone.0328893 (PMC12367152; doi:10.1371/journal.pone.0328893)

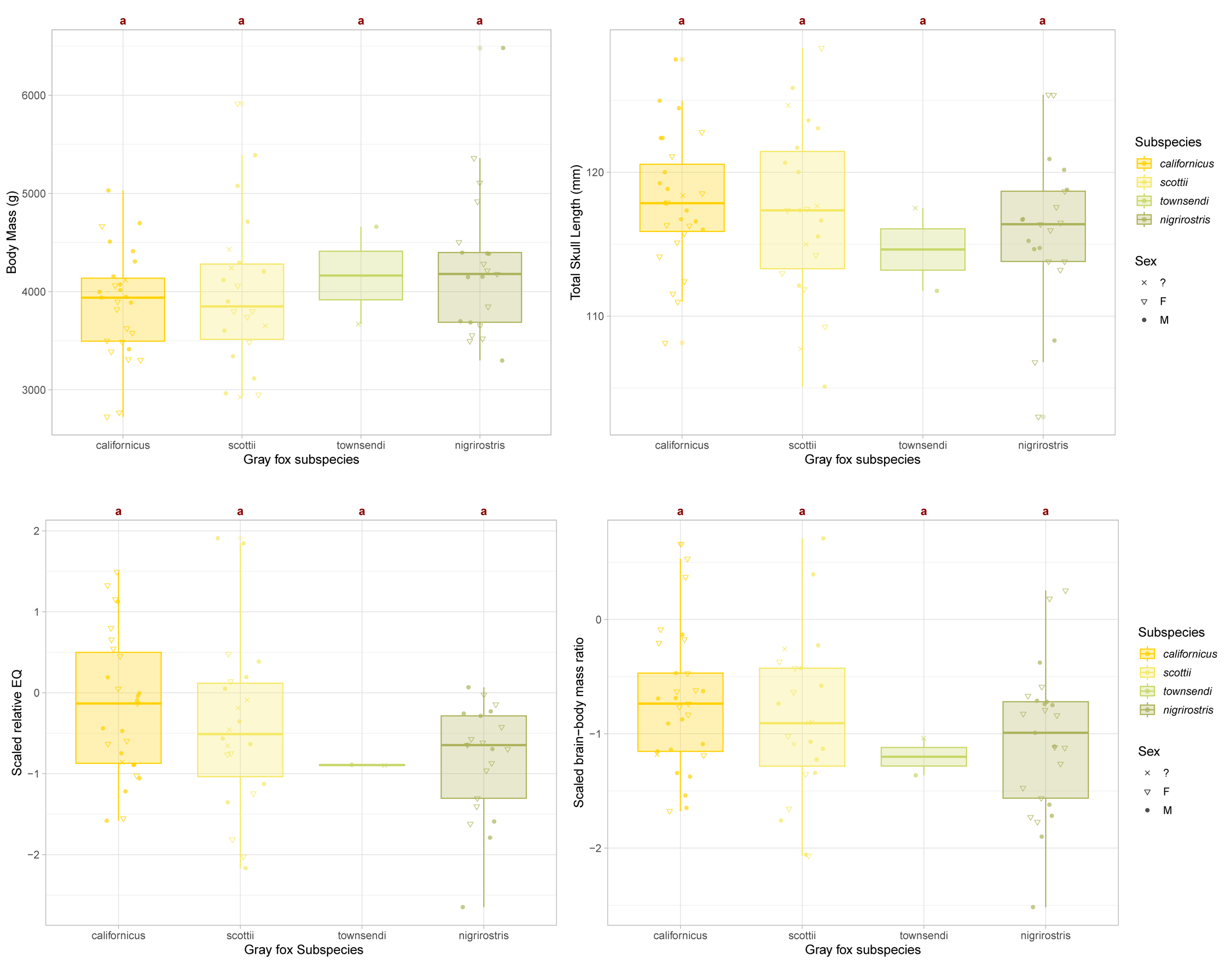

Supplement: S1 Fig — Means in all plots share the same letter, indicating no significant difference by Tukey-test at 5% level of significance. (TIF) [file pone.0328893.s001.tif]

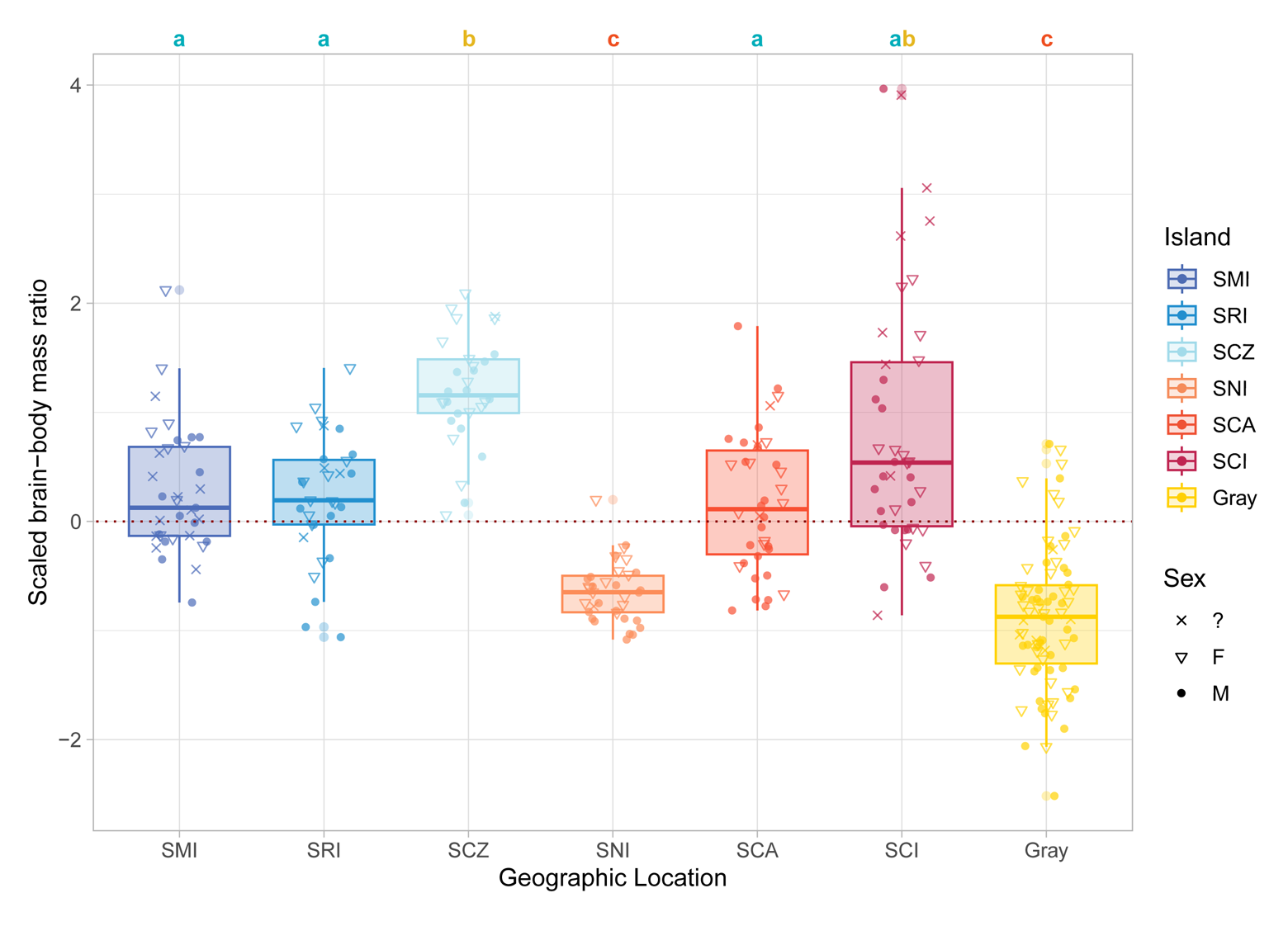

Supplement: S2 Fig — Values scale normalized to zero. Means not sharing any letter are significantly different by Dunnett’s T3 test at 5% level of significance. (TIF) [file pone.0328893.s002.tif]

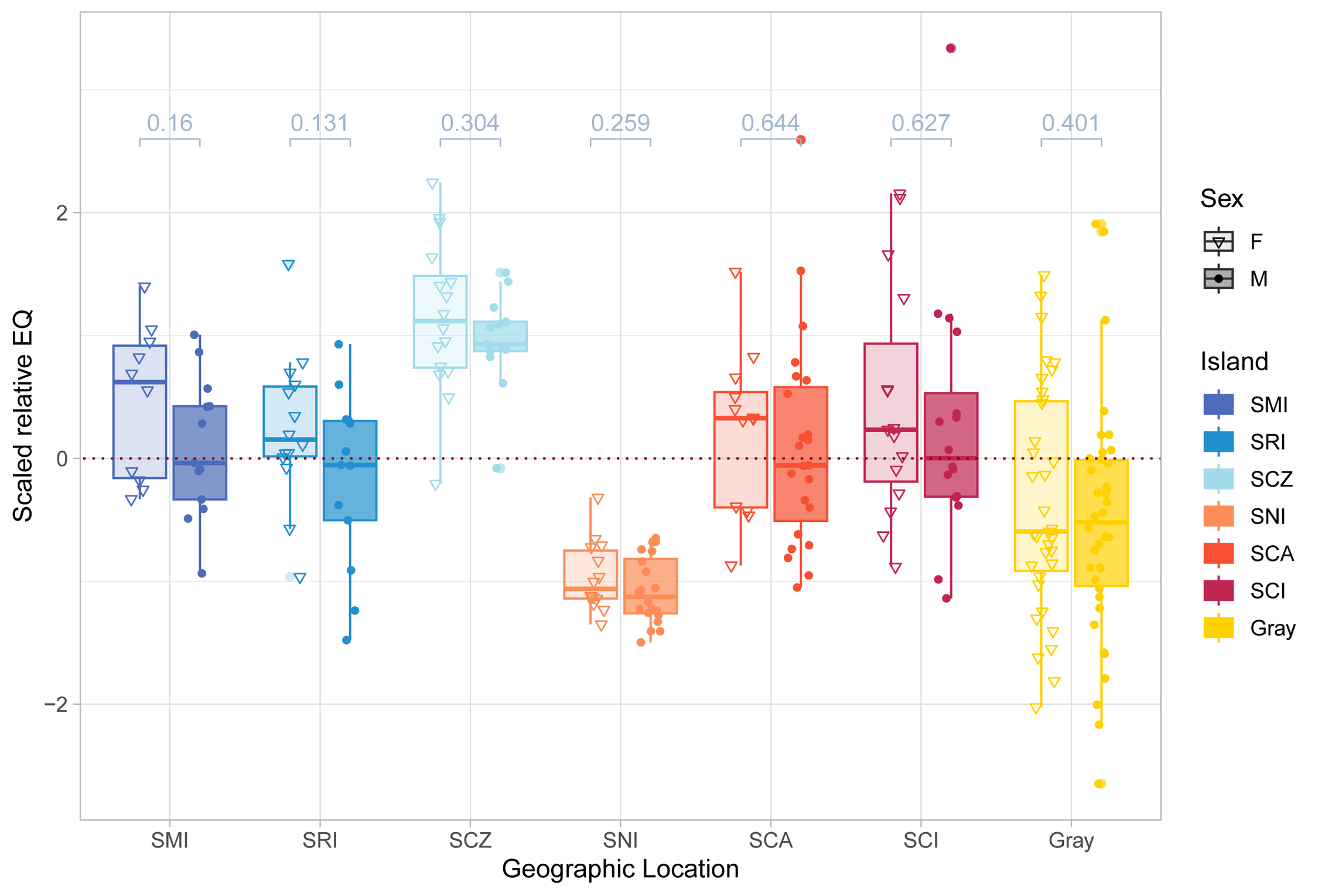

Supplement: S3 Fig — EQ values scale normalized to zero. Within group p-values for Tukey statistical differences shown in brackets above each pairing. (TIF) [file pone.0328893.s003.tif]
