# Supplementary material for: Increased brain size of the dwarf Channel Island fox (Urocyon littoralis) challenges “Island Syndrome” and suggests little evidence of domestication
Source: PLoS One. 2025 Aug 20;20(8):e0328893. doi: 10.1371/journal.pone.0328893 (PMC12367152; doi:10.1371/journal.pone.0328893)
Supplement: S2 Table — Manual volumes were measured via bead displacement, digital volumes measured using endocast segmentation and surface volumes in Avizo. (PDF) [file pone.0328893.s005.pdf]

						Volumes (cm3)		
						Physical	Avizo	
**SpecimenNo**	**Collection**	**Age**	**Species**	**Subspecies**	**Sex**	**ECV**	**ECV (digital)**	**Difference**
8365	NHM	Adult	*cinereoargenteus*	*californicus*	M	45.5	45.30	0.20
8143	NHM	Adult	*cinereoargenteus*	*californicus*	M	37.0	36.34	0.66
22370	NHM	Adult	*cinereoargenteus*	*californicus*	M	38.5	37.54	0.96
52252	NHM	Adult	*cinereoargenteus*	*californicus*	M	42.0	42.53	0.53
87421	NHM	Adult	*cinereoargenteus*	*californicus*	M	42.0	42.03	0.03
7958	NHM	Adult	*littoralis*	*catalinae*	M	31.0	31.43	0.43
7965	NHM	Adult	*littoralis*	*catalinae*	M	31.0	30.81	0.19
74997	NHM	Adult	*littoralis*	*catalinae*	M	33.0	33.07	0.07
74999	NHM	Adult	*littoralis*	*catalinae*	M	31.0	30.43	0.57
75000	NHM	Adult	*littoralis*	*catalinae*	M	31.1	31.11	0.01
