# Supplementary material for: Increased brain size of the dwarf Channel Island fox (Urocyon littoralis) challenges “Island Syndrome” and suggests little evidence of domestication
Source: PLoS One. 2025 Aug 20;20(8):e0328893. doi: 10.1371/journal.pone.0328893 (PMC12367152; doi:10.1371/journal.pone.0328893)
Supplement: S3 Table — Comparisons above dashed lines indicate results from PCA of log10 raw linear measurements, comparisons below dashed lines indicate results from PCA of log10 normalized measurements. (PDF) [file pone.0328893.s006.pdf]

	SMI	SCZ	SRI	SCA	SCI	SNI	Gray
**SMI**	--	2.17E-09	0.00021	3.09E-08	2.46E-12	6.37E-09	1.26E-44
**SCZ**	2.99E-09	--	8.23E-05	4.56E-07	6.94E-09	1.31E-10	1.28E-46
**SRI**	8.80E-05	0.00012	--	9.66E-08	2.18E-08	3.67E-07	6.54E-45
**SCA**	2.95E-08	0.00013	1.56E-07	--	3.18E-12	1.38E-09	1.23E-36
**SCI**	3.41E-12	1.94E-09	2.31E-08	2.85E-10	--	4.27E-14	1.75E-54
**SNI**	3.81E-09	4.08E-11	1.58E-07	1.69E-08	1.17E-14	--	8.68E-47
**Gray**	6.11E-33	3.43E-33	2.11E-35	5.92E-24	1.43E-40	5.91E-31	--
