# Supplementary material for: Increased brain size of the dwarf Channel Island fox (Urocyon littoralis) challenges “Island Syndrome” and suggests little evidence of domestication
Source: PLoS One. 2025 Aug 20;20(8):e0328893. doi: 10.1371/journal.pone.0328893 (PMC12367152; doi:10.1371/journal.pone.0328893)
Supplement: S1 Appendix — (PDF) [file pone.0328893.s007.pdf]

```r
#__Island Fox Encephalization R Code -- EQ and PCA__ 2025

#SETUP
#------

#Description of package use
#--------------------------------------------------------
#devtools used for general R functions
#readxl used to import raw data (read_xl)
#ggplot2 used for visualization and plots
#ggpubr used with ggplot2 for ggboxplot
#ggfortify used with ggplot2 for autoplot of PCA prcomps
#ggcorrplot used with ggplot2 for correlation plots and analyses
#tidyverse used for data manipulation ("%>%")
#rstatix used for statistical tests w/tidyverse (wilcox_test)
#corrplot used for correlation studies (corrplot)
#reshape2 used for correlation organization (melt)
#dplyr used for data organization (mutate_all)
#ggalt used for polygon clustering in ggplot (geom_encircle)
#factoextra used in PCA for visualizing eigenvectors
#multcomp used for parametric testing (glht)
#multcompView used for compact letter display significance
#rnaturalearth, rnaturalearthdata, and rnaturalearthhires for creating maps
#sf used for generating lat and long coordinate systems
#ggspatial used to generate scalebar on map (annotate_scale)
#car used for leveneTest
#PMCMR plus used to assess groupMeans
#marmap used to generate bathymetry map for Santarosae


#Package setup
packages = c("devtools", "readxl", "ggplot2", "ggpubr", "ggfortify","tidyverse",
             "rstatix", "reshape2", "dplyr", "ggcorrplot", "corrplot", "ggalt",
             "factoextra", "multcomp", "multcompView", "rnaturalearth",
             "rnaturalearthdata", "rnaturalearthhires", "sf", "car",
             "PMCMRplus", "ggspatial", "marmap")
lapply(packages, library, character.only = TRUE)

#Setting working directory
setwd("/Working_Directory")

#Importing data
#Excel Document with linear measurements and specimens
#NOTE: This data is from tab 1 of the S1 Table.xlsx
raw.dat = readexcel("2025_Urocyon Specimens and Measurements_Complete.xlsx")

#Excel Document with geographic coordinates for gray fox specimens
#NOTE: This data is from tab 2 of the S1 Table.xlsx, but written
#here as a separate excel document for ease of notation
g.coord = readexcel("2025_Urocyon_Gray Fox_Location Info.xlsx")

#Changing working directory for exports
setwd("/Exports")


#Generating Formulas and modifying datasets
#-------------------------------------------

#Functions:
#Calculating body mass from occipital condylar width
BM.fun = function(ocw) exp(8.5852*(log(ocw)^(2/3)) - 10.2696) #carnivora only
#Expected encephalization from body mass
E.EQ.fun = function(BM) 0.12*(BM^(2/3))
#Encephalization quotient from E.EQ and actual endocranial volume
```

```
EQ.fun = function(ECV,E.EQ) ECV/E.EQ
#Defining min-max normalization for measurements
minmax.fun = function(x) (x - min(x)) / (max(x) - min(x))
#Brain to body mass percentage ratio (use to compare to dogs/cats/etc)
BBMR.fun = function(ECV, BM) 100*(ECV/BM)


#Calculating values for all specimens
BM = BM.fun(raw.dat$OCW)
E.EQ = E.EQ.fun(BM)
EQ = EQ.fun(raw.dat$ECV, E.EQ)

#Appending calculated values to original data
fox.base = cbind(raw.dat, BM, E.EQ, EQ)
fox.data = data.frame(na.omit(fox.base))
fox.data$Island = factor(fox.data$Island, levels=c("SMI", "SRI", "SCZ", "SNI",
                                                   "SCA", "SCI", "Gray"))
fox.data$Subspecies = factor(fox.data$Subspecies,
                             levels=c("littoralis", "santarosae", "santacruzae",
                                      "dickeyi", "catalinae", "clementae",
                                      "californicus", "scottii", "townsendi",
                                      "nigrirostris"))

g.coord$Subspecies = factor(g.coord$Subspecies,
                             levels=c("californicus", "scottii", "townsendi",
                                      "nigrirostris"))

#Generating normalized dataset with MINMAX
fox.minmax = fox.data %>%
  mutate(across(where(is.numeric), minmax.fun))

#Calculating bbmr for all specimens
BBMR = BBMR.fun(fox.data$ECV, fox.data$BM)

#Appending calculated values to original data
fox.bbmr = cbind(fox.data, BBMR)

#Graphics settings
#-----------------
#Setting colors and shapes
loc.col = c("#d3d448","#fc8d59", "#2290cf", "#ffd100")
isl.col = c("#4c6cb9","#2290cf","#a2dceb","#fc8d59","#f85033","#c02550","#ffd100")
spec.col = c("#ffd100", "#7451a5")
subspec.col = c("#4c6cb9","#2290cf","#a2dceb","#fc8d59","#f85033","#c02550",
                "#ffd100", "#F5E864", "#C8D768", "#ADB35C")
sex.sh2 = c(6, 16)
sex.sh = c(4, 6, 16)
age.sh = c(18, 1)
spec.sh = c(18, 17)


#Subsetting data
#-----------------

#-- Main --
#All gray specimens
gray.data = fox.data[fox.data$Species == "cinereoargenteus",]
#All island specimens
isl.data = fox.data[fox.data$Species == "littoralis",]
#All adults
fox.adult = fox.data[fox.data$Age == "Adult", ]
#All adults NORMALIZED
fox.ad.norm = fox.minmax[fox.minmax$Age == "Adult", ]
#Gray adults NORMALIZED
g.fox.ad.norm = fox.ad.norm[fox.ad.norm$Species == "cinereoargenteus",]
```

```
#Island adults NORMALIZED
i.fox.ad.norm = fox.ad.norm[fox.ad.norm$Species == "littoralis",]


#-- By Age --
#Gray adults
gray.adult = gray.data[gray.data$Age == "Adult", ]
#Island adults
isl.adult = isl.data[isl.data$Age == "Adult", ]
#Gray juveniles
gray.juv = gray.data[gray.data$Age == "Juvenile", ]
#All island adults vs. gray juveniles
isl.vs.juv = rbind(isl.adult, gray.juv)

#Normalizing island adult vs. gray juveniles with MINMAX
ia.gj.minmax = isl.vs.juv %>%
  mutate(across(where(is.numeric), minmax.fun))

# -- Generating averages for ECV and BM of adult specimens per group --

#By island and gray
EQ.mean.summary = fox.adult %>%
  group_by(Island) %>%
  summarise_at(c("ECV", "BM", "EQ"), mean)

EQ.median.summary = fox.adult %>%
  group_by(Island) %>%
  summarise_at(c("ECV", "BM", "EQ"), median)

#By subspecies
EQ.mean.summary2 = fox.adult %>%
  group_by(Subspecies) %>%
  summarise_at(c("ECV", "BM", "EQ"), mean)

EQ.median.summary2 = fox.adult %>%
  group_by(Subspecies) %>%
  summarise_at(c("ECV", "BM", "EQ"), median)

#By species
#By island and gray
EQ.mean.summary3 = fox.adult %>%
  group_by(Species) %>%
  summarise_at(c("ECV", "BM", "EQ"), mean)

EQ.median.summary3 = fox.adult %>%
  group_by(Species) %>%
  summarise_at(c("ECV", "BM", "EQ"), median)

#Extracting gray subspecies
EQ.g.mean = EQ.mean.summary2[7:10,]
EQ.g.median = EQ.median.summary2[7:10,]



#--------------ENCEPHALIZATION---------------
#--------------------------------------------

#PART 1: ALLOMETRY
#------------------

# -- Model Setup and extraction of values from linear regressions --

#lm model pvalue extraction function
overall_p = function(model) {
  f = summary(model)$fstatistic
```

```
  p = pf(f[1],f[2],f[3],lower.tail=F)
  attributes(p) <- NULL
  return(p)
}

#significance annotation function
sig.fun = function(model) case_when(model >= 0.05 ~ "",
                                    model < 0.05 & model >= 0.01 ~ "< 0.05",
                                    model < 0.01 & model >= 0.001 ~ "< 0.01",
                                    model < 0.001 ~ "< 0.001")

r_squared_label = "R\u00B2" #generates unicode for R^2 for labels

#--Generating model and extracting values
mod = lm(log(ECV) ~ log(BM/1000), data = fox.adult) #formula for model
mod.int = mod[["coefficients"]][["(Intercept)"]] #obtains intercept from model
mod.slope = mod[["coefficients"]][["log(BM/1000)"]] #obtains slope from model
mod.pval = overall_p(mod) #calculates p value from model
mod.rsq = summary(mod)$r.squared #obtains R^2 value from model

#--Extracting models from each species--
#--Model 2: Island fox regression
mod2 = lm(log(ECV) ~ log(BM), data = subset(fox.adult, Species == "littoralis"))
mod2.slope = mod2[["coefficients"]][["log(BM)"]] #obtains slope from model
mod2.pval = overall_p(mod2) #calculates p value from model
mod2.rsq = summary(mod2)$r.squared #obtains R^2 value from model
mod2.se = coef(summary(mod2))["log(BM)", "Std. Error"] #std error from model
mod2.int = mod2[["coefficients"]][["(Intercept)"]]

#--Model 3: Gray fox regression
mod3 = lm(log(ECV) ~ log(BM), data = subset(fox.adult, Species == "cinereoargenteus"))
mod3.slope = mod3[["coefficients"]][["log(BM)"]] #obtains slope from model
mod3.pval = overall_p(mod3) #calculates p value from model
mod3.rsq = summary(mod3)$r.squared #obtains R^2 value from model
mod3.se = coef(summary(mod3))["log(BM)", "Std. Error"] #std error from model
mod3.int = mod3[["coefficients"]][["(Intercept)"]]

#--Model interactions: Checking for interactions between ECV and species from BM
mod.sp = lm(log(ECV) ~ log(BM)*Species, data = fox.adult)
an.mod.sp = anova(mod.sp) #running anova

#INTERCEPTS
#Extracting intercept for the reference species (here, gray fox)
msp.gray.int = mod.sp[["coefficients"]][["(Intercept)"]]
#Extracting difference in intercepts between island and gray fox
msp.int.diff = mod.sp[["coefficients"]][["Specieslittoralis"]]
#Extracting p value for intercept difference
msp.int.diff.pval = coef(summary(mod.sp))["Specieslittoralis", "Pr(>|t|)"]

#SLOPES
#Extracting slope for reference group
msp.gray.slope = mod.sp[["coefficients"]][["log(BM)"]]
#Extracting difference in slopes between island and gray fox
msp.slope.diff = mod.sp[["coefficients"]][["log(BM):Specieslittoralis"]]
#Extracting p value for slope difference
msp.slope.diff.pval = coef(summary(mod.sp))["log(BM):Specieslittoralis",
                                            "Pr(>|t|)"]

#COMBINING INTO ONE TABLE
#compiling values
reg.summary = rbind(mod2.slope, mod3.slope, msp.slope.diff, msp.slope.diff.pval,
                    mod2.int, mod3.int, msp.int.diff, msp.int.diff.pval)
#renaming rows
rownames(reg.summary) = c("Island fox slope", "Gray fox slope",
                          "Slope difference", "Slope difference pval",
```

```
                             "Island fox intercept", "Gray fox intercept",
                             "Intercept difference", "Intercept difference pval")


#--Extracting significance from linear regression models
allo.sig = sig.fun(mod.pval)
allo.sig2 = sig.fun(mod2.pval)
allo.sig3 = sig.fun(mod3.pval)


#Creating label(s) for plot
#Urocyon full
mod.lab = paste(r_squared_label," = ", round(mod.rsq, digits = 2), "\n",
                "p ", allo.sig, "\n",
                sep = "")
uslope.lab = paste("Urocyon:", round(mod.slope, digits = 2)) #extracts just slope

#Island fox model label
mod2.lab = paste(r_squared_label," = ", round(mod2.rsq, digits = 2), "\n",
                 "p ", allo.sig2, "\n",
                 "Slope = ", round(mod2.slope, digits = 2), "\n",
                 "Int = ", round(mod2.int, digits = 2), "\n",
                 sep = "")

#Gray fox model label
mod3.lab = paste(r_squared_label," = ", round(mod3.rsq, digits = 2), "\n",
                 "p ", allo.sig3, "\n",
                 "Slope = ", round(mod3.slope, digits = 2), "\n",
                 "Int = ", round(mod3.int, digits = 2), "\n",
                 sep = "")


# -- REGRESSION PLOT BODY MASS (g) VS ECV (cm^3) -- Fig3
#Encephalization slope clustered by species (slope) and subspecies (points)
ggplot(fox.adult) +
  aes(x = log(BM), y = log(ECV)) +
  geom_point(size = 2, aes(color = Subspecies, shape = Sex)) +
  theme(legend.position = "left") +
  xlab("Natural Log Body Mass (g)") +
  ylab(bquote("Natural Log Endocranial Volume"~cm^3)) +
  scale_colour_manual(values = subspec.col) +
  scale_shape_manual(values = sex.sh) +
  theme_light(base_size = 12) +
  #Regression line of canidae
  geom_abline(slope = 0.67, intercept = log(0.12), linetype = "dotted",
              color = "darkred") +
  #Regression line for each species
  stat_smooth(data = subset(fox.adult, Species == "cinereoargenteus"),
              aes(linetype = Species), method = "lm", formula = y ~ x,
              geom = "smooth", fill = NA, color = spec.col[1], linewidth=0.7) +
  stat_smooth(data = subset(fox.adult, Species == "littoralis"),
              aes(linetype = Species), method = "lm", formula = y ~ x,
              geom = "smooth", fill = NA, color = spec.col[2], linewidth=0.7) +
  scale_linetype_manual(values = c("solid", "solid")) +
  #Regression line for the whole plot
  stat_smooth(aes(group = 1), method = "lm", formula = y ~ x,
              geom = "smooth", fill = NA, linewidth = 0.5, color = "gray28",
              linetype = "longdash") +
  #Annotation with models regression info
  #All Urocyon
  annotate("text", x=8.55, y=3.12, hjust=0, label=mod.lab, color = "gray28") +
  #Island fox
  annotate("text", x=7.15, y=3.8, hjust=0, label=mod2.lab, color = "#7451a5") +
  #Gray fox
  annotate("text", x=7.4, y=3.8, hjust=0, label=mod3.lab, color = "#ffd100")
```

```
#Exporting plot
ggsave("Regression_BM-ECV.pdf", plot = last_plot(), device = NULL,
       path = NULL, scale = 0.65, width = NA, height = NA,
       units = "in", dpi = 300)



# -- REGRESSION PLOT SKULL LENGTH (MM) VS ECV (cm^3) -- Supplement

#Generating model and pulling data for labels
mod.TSL = lm(log(ECV) ~ log(TSL), data = fox.adult)
TSL.rsq = summary(mod.TSL)$r.squared #obtains R^2 value from model
allo.sig4 = sig.fun(mod.pval) #pvalue
mod.TSL.slope = mod.TSL[["coefficients"]][["log(TSL)"]] #obtains slope from model
mod.TSL.int = mod.TSL[["coefficients"]][["(Intercept)"]] #obtains slope from model

#Creating label for Urocyon full
mod.lab.TSL = paste(r_squared_label," = ", round(TSL.rsq, digits = 2), "\n",
                "p ", allo.sig4, "\n",
                sep = "")



#Encephalization slope clustered by species (slope) and subspecies (points)
ggplot(fox.adult) +
  aes(x = log(TSL), y = log(ECV)) +
  geom_point(size = 2, aes(color = Subspecies, shape = Sex)) +
  theme(legend.position = "left") +
  xlab("Natural Log Skull Length (mm)") +
  ylab(bquote("Natural Log Endocranial Volume"~cm^3)) +
  scale_colour_manual(values = subspec.col) +
  scale_shape_manual(values = sex.sh) +
  theme_light(base_size = 12) +
  #Regression line for each species
  stat_smooth(data = subset(fox.adult, Species == "cinereoargenteus"),
              aes(linetype = Species), method = "lm", formula = y ~ x,
              geom = "smooth", fill = NA, color = spec.col[1], linewidth=0.7) +
  stat_smooth(data = subset(fox.adult, Species == "littoralis"),
              aes(linetype = Species), method = "lm", formula = y ~ x,
              geom = "smooth", fill = NA, color = spec.col[2], linewidth=0.7) +
  scale_linetype_manual(values = c("solid", "solid")) +
  #Regression line for the whole plot
  stat_smooth(aes(group = 1), method = "lm", formula = y ~ x,
              geom = "smooth", fill = NA, linewidth = 0.5, color = "gray28",
              linetype = "longdash") +
  #Annotation with models regression info
  #All Urocyon
  annotate("text", x=4.8, y=3.12, hjust=0, label=mod.lab.TSL, color = "gray28")

#Exporting plot
ggsave("Regression_TSL-ECV.pdf", plot = last_plot(), device = NULL,
       path = NULL, scale = 0.65, width = NA, height = NA,
       units = "in", dpi = 300)

#--Checking for significant differences

#--Model interactions: between ECV and species from skull length
msp.TSL = lm(log(ECV) ~ log(TSL)*Species, data = fox.adult)

#--Getting island fox data
msp.TSL.i = lm(log(ECV) ~ log(TSL),
               data = subset(fox.adult, Species == "littoralis"))
msp.TSL.i.slope = msp.TSL.i[["coefficients"]][["log(TSL)"]] #obtains slope from model
msp.TSL.i.int = msp.TSL.i[["coefficients"]][["(Intercept)"]]
```

```
msp.TSL.i.pval = overall_p(msp.TSL.i) #calculates p value from model
msp.TSL.i.rsq = summary(msp.TSL.i)$r.squared #obtains R^2 value from model

#--Getting gray fox data
msp.TSL.g = lm(log(ECV) ~ log(TSL),
               data = subset(fox.adult, Species == "cinereoargenteus"))
msp.TSL.g.pval = overall_p(msp.TSL.g) #calculates p value from model
msp.TSL.g.rsq = summary(msp.TSL.g)$r.squared #obtains R^2 value from model


#INTERCEPTS
#Extracting intercept for the reference species (here, gray fox)
msp.TSL.gray.int = msp.TSL[["coefficients"]][["(Intercept)"]]
#Extracting difference in intercepts between island and gray fox
msp.TSL.int.diff = msp.TSL[["coefficients"]][["Specieslittoralis"]]
#Extracting p value for intercept difference
msp.TSL.int.diff.pval = coef(summary(msp.TSL))["Specieslittoralis", "Pr(>|t|)"]

#SLOPES
#Extracting slope for reference group
msp.TSL.gray.slope = msp.TSL[["coefficients"]][["log(TSL)"]]
#Extracting difference in slopes between island and gray fox
msp.TSL.slope.diff = msp.TSL[["coefficients"]][["log(TSL):Specieslittoralis"]]
#Extracting p value for slope difference
msp.TSL.slope.diff.pval = coef(summary(msp.TSL))["log(TSL):Specieslittoralis",
                                            "Pr(>|t|)"]

#COMBINING INTO ONE TABLE
#compiling values
reg.summary2 = rbind(msp.TSL.i.slope, msp.TSL.gray.slope,
                     msp.TSL.slope.diff, msp.TSL.slope.diff.pval,
                     msp.TSL.i.int, msp.TSL.gray.int,
                     msp.TSL.int.diff, msp.TSL.int.diff.pval)
#renaming rows
rownames(reg.summary2) = c("Island fox slope", "Gray fox slope",
                          "Slope difference", "Slope difference pval",
                          "Island fox intercept", "Gray fox intercept",
                          "Intercept difference", "Intercept difference pval")




#PART 2: ADULT EQ BOXPLOT COMPARISON WITH P VALUES
#-------------------------------------------

#_____________ENCEPHALIZATION QUOTIENT_____________ --MAIN--

#--Setting up dataframe--
#Scaled EQ from data (mean normalized to 0)
EQ.df = fox.data %>%
  mutate(across(where(is.numeric), scale))
EQ.df$Island = as.factor(EQ.df$Island) #converting vector to a factor
EQ.df$Subspecies = as.factor(EQ.df$Subspecies) #converting vector to a factor
EQ.df$Species = as.factor(EQ.df$Species) #converting vector to a factor

#Subsetting gray fox adult normalized data
EQ.df.g = EQ.df[EQ.df$Species == "cinereoargenteus", ]
EQ.df.g = EQ.df.g[EQ.df.g$Age == "Adult", ]


#--BY SPECIES ONLY--

#Normality
#Shapiro-Wilk Normality Test by SPECIES
fox.adult %>%
```

```
  group_by(Species) %>%
  shapiro_test(EQ)
#Failed, proceed to Mann-Whitney

#Notation for this says "wilcox.test" but it is a Mann Whitney
spec.sig = wilcox.test(EQ ~ Species, fox.data) #there are sig differences
spec.p = spec.sig$p.value #extracting p-value


#Plotting raw EQ by Species
ggboxplot(fox.data, x = "Species", y = "EQ", color = "Species",
          fill = "Species", alpha = 0.3) +
  scale_fill_manual(values = rev(spec.col)) +
  scale_color_manual(values = rev(spec.col)) +
  scale_shape_manual(values = sex.sh) +
  geom_jitter(aes(color = Species, shape = Sex), alpha = 0.7, width = 0.2) +
  theme_light(base_size = 12) +
  xlab("Species") +
  ylab("Unscaled EQ") +
  #Adding significance test values
  geom_bracket(xmin = "littoralis", xmax = "cinereoargenteus", y.position = 1.83,
               label = round(spec.p, digits = 8), tip.length = 0.01,
               color = "lightsteelblue3") +
  #Adding a dashed line for the mean
  stat_summary(geom = "errorbar", fun.min = mean, fun = mean, fun.max = mean,
               width = 0.7, color = rev(spec.col), linetype = "dotted") +
  #Add text labels for mean values
  annotate("text", x = c(2, 1), y = Inf, vjust = 2,
           label = round(EQ.mean.summary3$EQ, digits = 2),
           size = 4, color = spec.col)

#Exporting plot
ggsave("Box-unscaled-EQ_Species.pdf", plot = last_plot(), device = NULL,
       path = NULL, scale = 0.65, width = NA, height = NA,
       units = "in", dpi = 300)



#BY ISLAND

#--Testing for Normality and Variance--
#Shapiro-Wilk Normality Test by ISLAND
fox.data %>%
  group_by(Island) %>%
  shapiro_test(EQ)
#All passed and appear normally distributed but SCI


#SCI p = 0.037, subsetting and checking QQplot
clem.sub = subset(fox.data, Island == "SCI")
ggqqplot(clem.sub$EQ)
#Data appear very close to normally distributed and has large sample size,
#proceeding with tests assuming normality

#--Checking for equal variance

#Levene Test to compare variance
leveneTest(EQ ~ Island, fox.data)
#p is significant, variance is not equal between groups

#--Testing if there are significant differences between groups--

#Performing ANOVA for island subspecies vs. gray
#Method 1: One way ANOVA
oneway.test(EQ ~ Island, data = fox.data, var.equal = FALSE)
```

```
#Method 2: General ANOVA
aov.isl = aov(EQ ~ Island, data = fox.data)
summary(aov.isl)

#p is significant for both, there are differences

#Dunnett T3 Test - posthoc
T3.isl = dunnettT3Test(EQ ~ Island, data = fox.data)
T3.pval = T3.isl$p.value
#Summary of letters from T3 test - note and add manually to plot
summaryGroup(T3.isl)


## Plotting EQ Values ##

#COMPARING ISLAND SUBSPECIES TO GRAY FOX --
#_Unscaled with annotated mean values_
ggboxplot(fox.data, x = "Island", y = "EQ", color = "Island",
          fill = "Island", alpha = 0.3) +
  scale_fill_manual(values = isl.col) +
  scale_color_manual(values = isl.col) +
  scale_shape_manual(values = sex.sh) +
  geom_jitter(aes(color = Island, shape = Sex), alpha = 0.7, width = 0.2) +
  theme_light(base_size = 12) +
  xlab("Geographic Location") +
  ylab("Unscaled EQ") +
  #Adding a dashed line for the mean
  stat_summary(geom = "errorbar", fun.min = mean, fun = mean, fun.max = mean,
               width = 0.7, color = isl.col, linetype = "dotted") +
  #Add text labels for mean values
  annotate("text", x = EQ.mean.summary$Island, y = Inf, vjust = 2,
           label = round(EQ.mean.summary$EQ, digits = 2),
           size = 4, color = isl.col)

ggsave("Box-unscaled-EQ_Island.pdf", plot = last_plot(), device = NULL,
       path = NULL, scale = 0.65, width = NA, height = NA,
       units = "in", dpi = 300)

#_Normalized to zero_
ggboxplot(EQ.df, x = "Island", y = "EQ", color = "Island",
          fill = "Island", alpha = 0.3) +
  scale_fill_manual(values = isl.col) +
  scale_color_manual(values = isl.col) +
  scale_shape_manual(values = sex.sh) +
  geom_jitter(aes(color = Island, shape = Sex), alpha = 0.7, width = 0.2) +
  theme_light(base_size = 12) +
  xlab("Geographic Location") +
  ylab("Scaled relative EQ") +
  geom_abline(slope = 0, intercept = 0, linetype = "dotted",
              color = "darkred") +
  #Adding a dashed line for the mean
  stat_summary(geom = "errorbar", fun.min = mean, fun = mean, fun.max = mean,
               width = 0.7, color = isl.col, linetype = "dotted")

ggsave("Box-scaled-EQ_Island.pdf", plot = last_plot(), device = NULL,
       path = NULL, scale = 0.65, width = NA, height = NA,
       units = "in", dpi = 300)


## EQ BONUS TEST GROUPS -- SUPPLEMENT --

#Comparing all subspecies
oneway.test(EQ ~ Subspecies, data = fox.data, var.equal = FALSE)
res.aov.ssp = aov(EQ ~ Subspecies, data = fox.data) #calculating residuals
```

```
#p is significant, there are differences

#Comparing gray subspecies
oneway.test(EQ ~ Subspecies, data = gray.adult, var.equal = FALSE)
res.aov.g = aov(EQ ~ Subspecies, data = gray.adult) #calculating residuals
#p is significant, there are differences

#Tukey test
#Summary Tukey tests by island and by subspecies (V1)
tuk.isl.sum = glht(aov.isl, linfct = mcp(Island = "Tukey")) #island and gray
tuk.ssp.sum = glht(res.aov.ssp, linfct = mcp(Subspecies = "Tukey")) #all ssp
tuk.g.sum = glht(res.aov.g, linfct = mcp(Subspecies = "Tukey")) #gray ssp


#Tukey tests and letters by island and by subspecies
#BY ISLAND
tuk.isl = TukeyHSD(res.aov.isl <- aov(EQ ~ Island, data = fox.data)) #tuk test
tuk.isl.let = multcompLetters(tuk.isl$Island[,4]) #generating letters
plab = multcompLetters2(EQ ~ Island, tuk.isl$Island[,"p adj"], fox.data)
plab.let = data.frame(plab$Letters) #extracting letters
plab.let = plab.let %>%
  rownames_to_column(var = "Island") #giving column 1 a title

#BY SUBSPECIES
tuk.ssp = TukeyHSD(res.aov.ssp <- aov(EQ ~ Subspecies, data = fox.data)) #tuk test
tuk.ssp.let = multcompLetters(tuk.ssp$Subspecies[,4]) #generating letters
plab2 = multcompLetters2(EQ ~ Subspecies, tuk.ssp$Subspecies[,"p adj"], fox.data)
plab.let2 = data.frame(plab2$Letters) #extracting letters
plab.let2 = plab.let2 %>%
  rownames_to_column(var = "Subspecies") #giving column 1 a title

#GRAY SUBSPECIES
tuk.g = TukeyHSD(res.aov.g <- aov(EQ ~ Subspecies, data = gray.adult)) #tuk test
tuk.g.let = multcompLetters(tuk.g$Subspecies[,4]) #generating letters
plab3 = tuk.g.let
plab.let3 = data.frame(plab3$Letters) #extracting letters
plab.let3 = plab.let3 %>%
  rownames_to_column(var = "Subspecies") #giving column 1 a title


#--Creating box plots--

#-unscaled with annotated mean values-

#COMPARING ISLAND SUBSPECIES TO GRAY FOX --
#_Unscaled with annotated mean values_
ggboxplot(fox.data, x = "Island", y = "EQ", color = "Island",
          fill = "Island", alpha = 0.3) +
  scale_fill_manual(values = isl.col) +
  scale_color_manual(values = isl.col) +
  scale_shape_manual(values = sex.sh) +
  geom_jitter(aes(color = Island, shape = Sex), alpha = 0.7, width = 0.2) +
  theme_light(base_size = 12) +
  xlab("Geographic Location") +
  ylab("Unscaled EQ") +
  #Adding a dashed line for the mean
  stat_summary(geom = "errorbar", fun.min = mean, fun = mean, fun.max = mean,
               width = 0.7, color = isl.col, linetype = "dotted") +
  #Adding Tukey letters
  annotate("text", x = plab.let$Island, y = Inf, vjust = 4,
           label = plab.let$plab.Letters,
           size = 4, color = "darkred") +
  #Add text labels for mean values
  annotate("text", x = EQ.mean.summary$Island, y = Inf, vjust = 2,
           label = round(EQ.mean.summary$EQ, digits = 2),
```

```
          size = 4, color = isl.col)

#Exporting plot
ggsave("Box-unscaled-EQ_Island.pdf", plot = last_plot(), device = NULL,
       path = NULL, scale = 0.65, width = NA, height = NA,
       units = "in", dpi = 300)

#_Normalized to zero_
ggboxplot(EQ.df, x = "Island", y = "EQ", color = "Island",
          fill = "Island", alpha = 0.3) +
  scale_fill_manual(values = isl.col) +
  scale_color_manual(values = isl.col) +
  scale_shape_manual(values = sex.sh) +
  geom_jitter(aes(color = Island, shape = Sex), alpha = 0.7, width = 0.2) +
  theme_light(base_size = 12) +
  xlab("Geographic Location") +
  ylab("Scaled relative EQ") +
  geom_abline(slope = 0, intercept = 0, linetype = "dotted",
              color = "darkred") +
  #Adding a dashed line for the mean
  stat_summary(geom = "errorbar", fun.min = mean, fun = mean, fun.max = mean,
               width = 0.7, color = isl.col, linetype = "dotted") +
  #Adding Tukey letters
  annotate("text", x = plab.let$Island, y = Inf, vjust = 4,
           label = plab.let$plab.Letters,
           size = 4, color = "darkred")

ggsave("Box-scaled-EQ_Island.pdf", plot = last_plot(), device = NULL,
       path = NULL, scale = 0.65, width = NA, height = NA,
       units = "in", dpi = 300)



#COMPARING ALL SUBSPECIES
#_Unscaled with annotated mean values_
ggboxplot(fox.data, x = "Subspecies", y = "EQ", color = "Subspecies",
          fill = "Subspecies", alpha = 0.3) +
  scale_fill_manual(values = subspec.col) +
  scale_color_manual(values = subspec.col) +
  scale_shape_manual(values = sex.sh) +
  geom_jitter(aes(color = Subspecies, shape = Sex), alpha = 0.7, width = 0.2) +
  theme_light(base_size = 12) +
  xlab("Subspecies") +
  ylab("Unscaled EQ") +
  #Adding a dashed line for the mean
  stat_summary(geom = "errorbar", fun.min = mean, fun = mean, fun.max = mean,
               width = 0.7, color = subspec.col, linetype = "dotted") +
  #Adding Tukey letters
  annotate("text", x = plab.let2$Subspecies, y = Inf, vjust = 4,
           label = plab.let2$plab2.Letters,
           size = 4, color = "darkred") +
  #Add text labels for mean values
  annotate("text", x = EQ.mean.summary2$Subspecies, y = Inf, vjust = 2,
           label = round(EQ.mean.summary2$EQ, digits = 2),
           size = 4, color = subspec.col)

ggsave("Box-unscaled-EQ_ssp.pdf", plot = last_plot(), device = NULL,
       path = NULL, scale = 0.65, width = NA, height = NA,
       units = "in", dpi = 300)

#_Normalized to zero_
ggboxplot(EQ.df, x = "Subspecies", y = "EQ", color = "Subspecies",
          fill = "Subspecies", alpha = 0.3) +
  scale_fill_manual(values = subspec.col) +
  scale_color_manual(values = subspec.col) +
```

```
  scale_shape_manual(values = sex.sh) +
  geom_jitter(aes(color = Subspecies, shape = Sex), alpha = 0.7, width = 0.2) +
  theme_light(base_size = 12) +
  xlab("Subspecies") +
  ylab("Scaled relative EQ") +
  geom_abline(slope = 0, intercept = 0, linetype = "dotted",
              color = "darkred") +
  #Adding a dashed line for the mean
  stat_summary(geom = "errorbar", fun.min = mean, fun = mean, fun.max = mean,
               width = 0.7, color = subspec.col, linetype = "dotted") +
  #Adding Tukey letters
  annotate("text", x = plab.let2$Subspecies, y = Inf, vjust = 4,
           label = plab.let2$plab2.Letters,
           size = 4, color = "darkred")

ggsave("Box-scaled-EQ_ssp.pdf", plot = last_plot(), device = NULL,
       path = NULL, scale = 0.65, width = NA, height = NA,
       units = "in", dpi = 300)


#COMPARING GRAY SUBSPECIES
#_Unscaled with annotated mean values_
ggboxplot(gray.adult, x = "Subspecies", y = "EQ", color = "Subspecies",
          fill = "Subspecies", alpha = 0.3) +
  scale_fill_manual(values = subspec.col[7:10]) +
  scale_color_manual(values = subspec.col[7:10]) +
  scale_shape_manual(values = sex.sh) +
  geom_jitter(aes(color = Subspecies, shape = Sex), alpha = 0.7, width = 0.2) +
  theme_light(base_size = 12) +
  xlab("Subspecies") +
  ylab("Unscaled EQ") +
  #Adding a dashed line for the mean
  stat_summary(geom = "errorbar", fun.min = mean, fun = mean, fun.max = mean,
               width = 0.7, color = subspec.col[7:10], linetype = "dotted") +
  #Adding Tukey letters
  annotate("text", x = plab.let3$Subspecies, y = Inf, vjust = 4,
           label = plab.let3$plab3.Letters,
           size = 4, color = "darkred") +
  #Add text labels for mean values
  annotate("text", x = EQ.g.mean$Subspecies, y = Inf, vjust = 2,
           label = round(EQ.g.mean$EQ, digits = 2),
           size = 4, color = subspec.col[7:10])

ggsave("Box-unscaled-EQ_ssp-GRAY.pdf", plot = last_plot(), device = NULL,
       path = NULL, scale = 0.65, width = NA, height = NA,
       units = "in", dpi = 300)

#_Normalized to zero_
ggboxplot(EQ.df.g, x = "Subspecies", y = "EQ", color = "Subspecies",
          fill = "Subspecies", alpha = 0.3) +
  scale_fill_manual(values = subspec.col[7:10]) +
  scale_color_manual(values = subspec.col[7:10]) +
  scale_shape_manual(values = sex.sh) +
  geom_jitter(aes(color = Subspecies, shape = Sex), alpha = 0.7, width = 0.2) +
  theme_light(base_size = 12) +
  xlab("Subspecies") +
  ylab("Scaled relative EQ") +
  geom_abline(slope = 0, intercept = 0, linetype = "dotted",
              color = "darkred") +
  #Adding a dashed line for the mean
  stat_summary(geom = "errorbar", fun.min = mean, fun = mean, fun.max = mean,
               width = 0.7, color = subspec.col[7:10], linetype = "dotted") +
  #Adding Tukey letters
  annotate("text", x = plab.let3$Subspecies, y = Inf, vjust = 4,
           label = plab.let3$plab3.Letters,
```

```
          size = 4, color = "darkred")

ggsave("Box-scaled-EQ_ssp-GRAY.pdf", plot = last_plot(), device = NULL,
       path = NULL, scale = 0.65, width = NA, height = NA,
       units = "in", dpi = 300)

#___Separating by SEX___

#BY ISLAND

#Subsetting to remove unknown sex
EQ.df.sex = EQ.df[EQ.df$Sex != "?",]

#Running t test to check for significant differences in means between M&F
EQsex.p = EQ.df.sex %>%
  group_by(Island) %>%
  t_test(EQ ~ Sex, ref.group = "M")
#adding XY coordinates info for plot
EQsex.p = EQsex.p %>% add_xy_position(x = "Island")


#Plotting boxplots by island with gray AND BY SEX -- scaled to zero
ggboxplot(EQ.df.sex, x = "Island", y = "EQ", color = "Island", shape = "Sex",
          fill = "Island", alpha = "Sex") +
  scale_fill_manual(values = isl.col) +
  scale_color_manual(values = isl.col) +
  scale_shape_manual(values = sex.sh[2:3]) +
  scale_alpha_manual(values=c(0.2, 0.7)) +
  geom_point(aes(color = Island, shape = Sex),
             position = position_jitterdodge(jitter.width = 0.7)) +
  theme_light(base_size = 12) +
  xlab("Geographic Location") +
  ylab("Scaled relative EQ") +
  stat_pvalue_manual(EQsex.p, label = "p", tip.length = 0.01,
                     step.increase = 0, y.position = 2.6, hide.ns = FALSE,
                     color = "lightsteelblue3") +
  geom_abline(slope = 0, intercept = 0, linetype = "dotted",
              color = "darkred")

ggsave("Box-EQ_sex.pdf", plot = last_plot(), device = NULL,
       path = NULL, scale = 0.65, width = NA, height = NA,
       units = "in", dpi = 300)


#EQ BY SEX AND SUBSPECIES

#Subsetting to remove unknown sex
EQ.df.sex2 = EQ.df[EQ.df$Sex != "?",]
EQ.df.sex2 = EQ.df.sex2[EQ.df.sex2$Subspecies != "townsendi",]

#Running t test to check for significant differences in means between M&F
EQsex.p2 = EQ.df.sex2 %>%
  group_by(Subspecies) %>%
  t_test(EQ ~ Sex, ref.group = "M")
#adding XY coordinates info for plot
EQsex.p2 = EQsex.p2 %>% add_xy_position(x = "Subspecies")
EQsex.p2[9,"x"] = 9
EQsex.p2[9,"xmin"] = 9.2
EQsex.p2[9,"xmax"] = 8.8


#Plotting boxplots by island with gray AND BY SEX -- scaled to zero
ggboxplot(EQ.df.sex2, x = "Subspecies", y = "EQ", color = "Subspecies",
          shape = "Sex", fill = "Subspecies", alpha = "Sex") +
  scale_fill_manual(values = c(subspec.col[1:8], subspec.col[10])) +
```

```
  scale_color_manual(values = c(subspec.col[1:8], subspec.col[10])) +
  scale_shape_manual(values = sex.sh[2:3]) +
  scale_alpha_manual(values=c(0.2, 0.7)) +
  geom_point(aes(color = Subspecies, shape = Sex),
             position = position_jitterdodge(jitter.width = 0.7)) +
  theme_light(base_size = 12) +
  xlab("Subspecies") +
  ylab("Scaled relative EQ") +
  stat_pvalue_manual(EQsex.p2, label = "p", tip.length = 0.01,
                     step.increase = 0, y.position = 2.6, hide.ns = FALSE,
                     color = "lightsteelblue3") +
  geom_abline(slope = 0, intercept = 0, linetype = "dotted",
              color = "darkred")

ggsave("Box-EQ_sex_ssp.pdf", plot = last_plot(), device = NULL,
       path = NULL, scale = 0.65, width = NA, height = NA,
       units = "in", dpi = 300)



#___________BRAIN TO BODY MASS RATIO___________

# -- Brain size comparison of Normalized BBMR: ISLAND TO GRAY --

#Performing one way anova to check if there are sig differences\

#Method 1: One way ANOVA
oneway.test(BBMR ~ Island, data = fox.bbmr, var.equal = FALSE)

#Method 2: General ANOVA
aov.isl2 = aov(BBMR ~ Island, data = fox.bbmr)
summary(aov.isl2)

#p is significant for both, there are differences


#Dunnett T3 Test
T3.isl2 = dunnettT3Test(BBMR ~ Island, data = fox.bbmr)
T3.pval2 = T3.isl2$p.value
summaryGroup(T3.isl2) #extracting letters from T3 test -- add later


#Normalized BBMR
BBMR.df = fox.bbmr %>%
  mutate(across(where(is.numeric), scale))

BBMR.df$Island = as.factor(BBMR.df$Island) #converting vector to a factor

#Plotting brain to body mass (BBMR) boxplots by island with gray
ggboxplot(BBMR.df, x = "Island", y = "BBMR", color = "Island",
          fill = "Island", alpha = 0.3) +
  scale_fill_manual(values = isl.col) +
  scale_color_manual(values = isl.col) +
  scale_shape_manual(values = sex.sh) +
  geom_jitter(aes(color = Island, shape = Sex), alpha = 0.7, width = 0.2) +
  theme_light(base_size = 12) +
  xlab("Geographic Location") +
  ylab("Scaled brain-body mass ratio") +
  geom_abline(slope = 0, intercept = 0, linetype = "dotted",
              color = "darkred")

ggsave("Box-scaled-BBMR_island.pdf", plot = last_plot(), device = NULL,
       path = NULL, scale = 0.65, width = NA, height = NA,
       units = "in", dpi = 300)
```

```
# -- Brain size comparison of Normalized BBMR: GRAY SUBSPECIES --

#Extracting gray fox specimens
BBMR.df2 = BBMR.df[BBMR.df$Species == "cinereoargenteus",]
BBMR.df2$Subspecies = as.factor(BBMR.df2$Subspecies) #converting vector to a factor


#Running Tukey test
tuk.bb.g = TukeyHSD(exp_aov <- aov(BBMR ~ Subspecies, data = BBMR.df2))
tuk.bb.g.let = multcompLetters(tuk.bb.g$Subspecies[,4])

#Extracting letters
plab.bb.g.let2 = data.frame(tuk.bb.g.let)
plab.bb.g.let2 =  plab.bb.g.let2 %>%
  rownames_to_column(var = "Subspecies") #giving column 1 a title


#Plotting brain to body mass (BBMR) boxplots by gray subspecies
ggboxplot(BBMR.df2, x = "Subspecies", y = "BBMR", color = "Subspecies",
          fill = "Subspecies", alpha = 0.3) +
  scale_fill_manual(values = subspec.col[7:10]) +
  scale_color_manual(values = subspec.col[7:10]) +
  scale_shape_manual(values = sex.sh) +
  geom_jitter(aes(color = Subspecies, shape = Sex), alpha = 0.7, width = 0.2) +
  theme_light(base_size = 12) +
  xlab("Gray fox subspecies") +
  ylab("Scaled brain-body mass ratio") +
  geom_abline(slope = 0, intercept = 0, linetype = "dotted",
              color = "darkred") +
  #Adding Tukey letters
  annotate("text", x = plab.bb.g.let2$Subspecies, y = Inf, vjust = 4,
           label = plab.bb.g.let2$Letters,
           size = 4, color = "darkred")

ggsave("Box-scaled-BBMR_ssp-GRAY.pdf", plot = last_plot(), device = NULL,
       path = NULL, scale = 0.65, width = NA, height = NA,
       units = "in", dpi = 300)




#-------------PRINCIPAL COMPONENT ANALYSIS--------------
#-----------------------------------------------------


#Extracting linear data
lin.data = cbind(fox.adult$Island, fox.adult$Sex, fox.adult[10:20])
colnames(lin.data)[1] = "Island"
colnames(lin.data)[2] = "Sex"

#Removing outlier 261
lin.data = lin.data[-245, ]

#-- NOT NORMALIZED --

#Running stats tests to verify match with PAST

#Log transforming data
log.dat = cbind(lin.data[1:2], log10(lin.data[3:13]))

#Computing the covariance matrix
cov.mat = data.frame(cov(log.dat[3:13]))

#Generating the eigenvectors
```

```
eig.mat = eigen(cov.mat)

#Extracting the eigenvalue percent variance for each PC
eig.vals = eig.mat$values
percent.var = eig.mat$values / sum(eig.mat$values)

#Summary table
cov.sum = data.frame(cbind(eig.vals, percent.var))

#--match works, proceeding with PCA for plot

#Running PCA
ad.log.pca = prcomp(log.dat[3:13], center = TRUE, scale. = FALSE)


#-- NORMALIZED TO TSL --

#Normalizing data to TSL
norm.dat = cbind(lin.data[1:2], (lin.data[4:13]/lin.data[,3]))

#Log transforming data
log.dat2 = cbind(lin.data[1:2], log10(norm.dat[3:12]))

#Computing the covariance matrix
cov.mat2 = data.frame(cov(log.dat2[3:12]))

#Generating the eigenvectors
eig.mat2 = eigen(cov.mat2)

#Extracting the eigenvalue percent variance for each PC
eig.vals2 = eig.mat2$values
percent.var2 = eig.mat2$values / sum(eig.mat2$values)

#Summary table
cov.sum2 = data.frame(cbind(eig.vals2, percent.var2))

#--match works, proceeding with PCA for plot

#Running PCA
ad.log.pca2 = prcomp(log.dat2[3:12], center = TRUE, scale. = FALSE)


#Note: An outlier was identified in both the non-normalized and normalized data
#as such, this outlier was removed and the PCA was run again.

#___PCA PLOTS____

#Plotting PCA1
#Log adult PCA (no eigen) by subspecies
autoplot(ad.log.pca, data = lin.data,
         colour = "Island", shape = "Sex",
         label = FALSE, frame = TRUE, loadings = FALSE,
         loadings.label = FALSE, loadings.label.size = 3.5,
         loadings.color = "#E3144B",
         loadings.label.hjust = 1.2, loadings.label.color = "#E3144B") +
  scale_shape_manual(values = sex.sh) +
  scale_colour_manual(values = isl.col) +
  scale_fill_manual(values = isl.col) +
  ggtitle(~italic("Urocyon:")~" Log PCA of adults by Island") +
  theme(plot.title = element_text(vjust = -1, size = 12)) +
  theme_light(base_size = 12) +
  geom_hline(yintercept = 0, linetype = "dashed", alpha = 0.3) +
  geom_vline(xintercept = 0, linetype = "dashed", alpha = 0.3)

ggsave("PCA_Log-Raw_ISLAND-Jun13_wide.pdf", plot = last_plot(), device = NULL,
```

```
       path = NULL, scale = 0.65, width = NA, height = NA,
       units = "in", dpi = 300)



#Plotting PCA2
#Log adult PCA (no eigen) by subspecies -- scaled to TSL
autoplot(ad.log.pca2, data = lin.data,
         colour = "Island", shape = "Sex",
         label = FALSE, frame = TRUE, loadings = FALSE,
         loadings.label = FALSE, loadings.label.size = 3.5,
         loadings.color = "#E3144B",
         loadings.label.hjust = 1.2, loadings.label.color = "#E3144B") +
  scale_shape_manual(values = sex.sh) +
  scale_colour_manual(values = isl.col) +
  scale_fill_manual(values = isl.col) +
  ggtitle(~italic("Urocyon:")~" Log PCA of adults by Island NORM") +
  theme(plot.title = element_text(vjust = -1, size = 12)) +
  theme_light(base_size = 12) +
  geom_hline(yintercept = 0, linetype = "dashed", alpha = 0.3) +
  geom_vline(xintercept = 0, linetype = "dashed", alpha = 0.3)

ggsave("PCA_Log-Norm_ISLAND-Jun13_wide.pdf", plot = last_plot(), device = NULL,
       path = NULL, scale = 0.65, width = NA, height = NA,
       units = "in", dpi = 300)


#___VARIABLE/EIGENVECTOR PLOTS___

#Loadings plot raw
fviz_pca_var(ad.log.pca, col.var="contrib",
             gradient.cols = c("#00AFBB", "#E7B800", "#FC4E07"),
             repel = TRUE,
             title = "Log-Raw PCA eigenvector contributions") +
  scale_x_continuous(expand=c(0, 0), limits=c(-0.04, 0.006)) +
  scale_y_continuous(expand=c(0, 0), limits=c(-0.023, 0.007))


ggsave("PCA_loadings_log-raw.pdf", plot = last_plot(), device = NULL,
       path = NULL, scale = 0.65, width = NA, height = NA,
       units = "in", dpi = 300)

#Loadings plot normalized
fviz_pca_var(ad.log.pca2, col.var="contrib",
             gradient.cols = c("#00AFBB", "#E7B800", "#FC4E07"),
             repel = TRUE,
             title = "Log-Normalized PCA eigenvector contributions") +
  theme_minimal()

ggsave("PCA_loadings_log-norm.pdf", plot = last_plot(), device = NULL,
       path = NULL, scale = 0.65, width = NA, height = NA,
       units = "in", dpi = 300)


#-------------BODY SIZE COMPARISON--------------
#---------------------------------------------


#--Testing for Normality and Variance--

#Removing gray fox subspecies townsendii for normality tests due to n = 2
fox.adult.tests = subset(fox.adult, Subspecies != "townsendi")

#TSL
#Shapiro-Wilk Normality Test
```

```
fox.adult.tests %>%
  group_by(Subspecies) %>%
  shapiro_test(TSL)
#Normally distributed, proceeding with parametric tests

#BM
#Shapiro-Wilk Normality Test
fox.adult.tests %>%
  group_by(Subspecies) %>%
  shapiro_test(BM)
#Two groups not normally distributed, checking subsets for outliers

#SCZ
cruz.sub = subset(fox.adult.tests, Island == "SCZ") #test subset
ggqqplot(cruz.sub$BM) #outlier identified
cruz.sub = subset(cruz.sub, SpecimenNo != "178") #removed Specimen 178
ggqqplot(cruz.sub$BM) #confirmed normal

#Nigrirostris
ni.sub = subset(fox.adult.tests, Subspecies == "nigrirostris") #test subset
ggqqplot(ni.sub$BM) #outlier identified
ni.sub = subset(ni.sub, SpecimenNo != "33262") #removed Specimen 33262
ggqqplot(ni.sub$BM) #confirmed normal

#Removing outliers from normality test dataset
fox.adult.tests = subset(fox.adult.tests, SpecimenNo != "178")
fox.adult.tests = subset(fox.adult.tests, SpecimenNo != "33262")

#Shapiro-Wilk Normality Test
fox.adult.tests %>%
  group_by(Subspecies) %>%
  shapiro_test(BM)
#Normally distributed, proceeding with parametric stats tests


#Adding back townsendi specimens
town.sub = subset(fox.adult, Subspecies == "townsendi")
fox.ad.size = rbind(fox.adult.tests, town.sub)
fox.ad.size$Subspecies = as.factor((fox.ad.size$Subspecies))

#--Testing if there are significant differences between groups--

#Performing Welch's ANOVA
#Comparing all subspecies for skull length
oneway.test(TSL ~ Subspecies, data = fox.ad.size, var.equal = FALSE)
res.aov.TSL = aov(TSL ~ Subspecies, data = fox.ad.size) #calculating residuals
#p is significant, there are differences

#Comparing all subspecies for body size
oneway.test(BM ~ Subspecies, data = fox.ad.size, var.equal = FALSE)
res.aov.BM = aov(BM ~ Subspecies, data = fox.ad.size) #calculating residuals
#p is significant, there are differences


#--Calculating statistical differences between groups (post-hoc)

#Tukey test summary
tuk.TSL.sum = glht(res.aov.TSL, linfct = mcp(Subspecies = "Tukey"))
tuk.BM.sum = glht(res.aov.BM, linfct = mcp(Subspecies = "Tukey"))

#Tukey tests and letters by subspecies
tuk.TSL = TukeyHSD(res.aov.TSL <- aov(TSL ~ Subspecies, data = fox.ad.size))
tuk.BM = TukeyHSD(res.aov.BM <- aov(BM ~ Subspecies, data = fox.ad.size))

#Generating Letters
```

```
TSL.lab = multcompLetters2(TSL ~ Subspecies, tuk.TSL$Subspecies[,"p adj"],
                           data = fox.ad.size)
TSL.let = data.frame(TSL.lab$Letters) #extracting letters
TSL.let = TSL.let %>%
  rownames_to_column(var = "Subspecies") #giving column 1 a title

BM.lab = multcompLetters2(BM ~ Subspecies, tuk.BM$Subspecies[,"p adj"],
                           data = fox.ad.size)
BM.let = data.frame(BM.lab$Letters) #extracting letters
BM.let = BM.let %>%
  rownames_to_column(var = "Subspecies") #giving column 1 a title


#--Plotting boxplots--
#TSL
ggboxplot(fox.ad.size, x = "Subspecies", y = "TSL", color = "Subspecies",
          fill = "Subspecies", alpha = 0.3) +
  scale_fill_manual(values = subspec.col) +
  scale_color_manual(values = subspec.col) +
  scale_shape_manual(values = sex.sh) +
  geom_jitter(aes(color = Subspecies, shape = Sex), alpha = 0.7, width = 0.2) +
  theme_light(base_size = 12) +
  xlab("Subspecies") +
  ylab("Total Skull Length (mm)") +
  #Adding a dashed line for the mean
  stat_summary(geom = "errorbar", fun.min = mean, fun = mean, fun.max = mean,
               width = 0.7, color = subspec.col, linetype = "dotted") +
  #Adding Tukey Letters
  annotate("text", x = TSL.let$Subspecies, y = Inf, vjust = 4,
           label = TSL.let$TSL.lab.Letters,
           size = 4, color = "darkred")

ggsave("Box_TSL_ssp.pdf", plot = last_plot(), device = NULL,
       path = NULL, scale = 0.65, width = NA, height = NA,
       units = "in", dpi = 300)

#BM
ggboxplot(fox.ad.size, x = "Subspecies", y = "BM", color = "Subspecies",
          fill = "Subspecies", alpha = 0.3) +
  scale_fill_manual(values = subspec.col) +
  scale_color_manual(values = subspec.col) +
  scale_shape_manual(values = sex.sh) +
  geom_jitter(aes(color = Subspecies, shape = Sex), alpha = 0.7, width = 0.2) +
  theme_light(base_size = 12) +
  xlab("Subspecies") +
  ylab("Body Mass (g)") +
  #Adding a dashed line for the mean
  stat_summary(geom = "errorbar", fun.min = mean, fun = mean, fun.max = mean,
               width = 0.7, color = subspec.col, linetype = "dotted") +
  #Adding Tukey Letters
  annotate("text", x = BM.let$Subspecies, y = Inf, vjust = 4,
           label = BM.let$BM.lab.Letters,
           size = 4, color = "darkred")

ggsave("Box_BM_ssp.pdf", plot = last_plot(), device = NULL,
       path = NULL, scale = 0.65, width = NA, height = NA,
       units = "in", dpi = 300)


#GRAY FOX ONLY

#Plotting TSL boxplots by gray subspecies
ggboxplot(gray.adult, x = "Subspecies", y = "TSL", color = "Subspecies",
          fill = "Subspecies", alpha = 0.3) +
  scale_fill_manual(values = subspec.col[7:10]) +
```

```
  scale_color_manual(values = subspec.col[7:10]) +
  scale_shape_manual(values = sex.sh) +
  geom_jitter(aes(color = Subspecies, shape = Sex), alpha = 0.7, width = 0.2) +
  theme_light(base_size = 12) +
  xlab("Gray fox subspecies") +
  ylab("Total Skull Length (mm)")

ggsave("Box_TSL_ssp_gray.pdf", plot = last_plot(), device = NULL,
       path = NULL, scale = 0.65, width = NA, height = NA,
       units = "in", dpi = 300)

#Plotting BM boxplots by gray subspecies
ggboxplot(gray.adult, x = "Subspecies", y = "BM", color = "Subspecies",
          fill = "Subspecies", alpha = 0.3) +
  scale_fill_manual(values = subspec.col[7:10]) +
  scale_color_manual(values = subspec.col[7:10]) +
  scale_shape_manual(values = sex.sh) +
  geom_jitter(aes(color = Subspecies, shape = Sex), alpha = 0.7, width = 0.2) +
  theme_light(base_size = 12) +
  xlab("Gray fox subspecies") +
  ylab("Body Mass (g)")

ggsave("Box_BM_ssp_gray.pdf", plot = last_plot(), device = NULL,
       path = NULL, scale = 0.65, width = NA, height = NA,
       units = "in", dpi = 300)


#___Body Mass separating by SEX___

#Subsetting to remove unknown sex
BM.df.sex = fox.ad.size[fox.ad.size$Sex != "?",]
BM.df.sex = BM.df.sex[BM.df.sex$Subspecies != "townsendi",]

#Running t test to check for significant differences in means between M&F
BMsex.p = BM.df.sex %>%
  group_by(Subspecies) %>%
  t_test(BM ~ Sex, ref.group = "M")
#adding XY coordinates info for plot
BMsex.p = BMsex.p %>% add_xy_position(x = "Subspecies")
#adjusting spacing for removing townsendii
BMsex.p[9,"x"] = 9
BMsex.p[9,"xmin"] = 9.2
BMsex.p[9,"xmax"] = 8.8

#Plotting body mass boxplots by subspecies AND BY SEX
ggboxplot(BM.df.sex, x = "Subspecies", y = "BM", color = "Subspecies",
          shape = "Sex", fill = "Subspecies", alpha = "Sex") +
  scale_fill_manual(values = c(subspec.col[1:8], subspec.col[10])) +
  scale_color_manual(values = c(subspec.col[1:8], subspec.col[10])) +
  scale_shape_manual(values = sex.sh[2:3]) +
  scale_alpha_manual(values=c(0.2, 0.7)) +
  geom_point(aes(color = Subspecies, shape = Sex),
             position = position_jitterdodge(jitter.width = 0.7)) +
  theme_light(base_size = 12) +
  xlab("Subspecies") +
  ylab("Body Mass (g)") +
  stat_pvalue_manual(BMsex.p, label = "p", tip.length = 0.01,
                     step.increase = 0, y.position = 5500, hide.ns = FALSE,
                     color = "lightsteelblue3")

ggsave("Box-BM_sex.pdf", plot = last_plot(), device = NULL,
       path = NULL, scale = 0.65, width = NA, height = NA,
       units = "in", dpi = 300)

#___TSL separating by SEX___
```

```
#Subsetting to remove unknown sex
TSL.df.sex = fox.ad.size[fox.ad.size$Sex != "?",]
TSL.df.sex = TSL.df.sex[TSL.df.sex$Subspecies != "townsendi",]

#Running t test to check for significant differences in means between M&F
TSLsex.p = TSL.df.sex %>%
  group_by(Subspecies) %>%
  t_test(TSL ~ Sex, ref.group = "M")
#adding XY coordinates info for plot
TSLsex.p = TSLsex.p %>% add_xy_position(x = "Subspecies")
#adjusting spacing for removing townsendii
TSLsex.p[9,"x"] = 9
TSLsex.p[9,"xmin"] = 9.2
TSLsex.p[9,"xmax"] = 8.8

#Plotting body mass boxplots by subspecies AND BY SEX
ggboxplot(TSL.df.sex, x = "Subspecies", y = "TSL", color = "Subspecies",
          shape = "Sex", fill = "Subspecies", alpha = "Sex") +
  scale_fill_manual(values = c(subspec.col[1:8], subspec.col[10])) +
  scale_color_manual(values = c(subspec.col[1:8], subspec.col[10])) +
  scale_shape_manual(values = sex.sh[2:3]) +
  scale_alpha_manual(values=c(0.2, 0.7)) +
  geom_point(aes(color = Subspecies, shape = Sex),
             position = position_jitterdodge(jitter.width = 0.7)) +
  theme_light(base_size = 12) +
  xlab("Subspecies") +
  ylab("Total Skull Length (mm)") +
  stat_pvalue_manual(TSLsex.p, label = "p", tip.length = 0.01,
                     step.increase = 0, y.position = 130, hide.ns = FALSE,
                     color = "lightsteelblue3")

ggsave("Box-TSL_sex.pdf", plot = last_plot(), device = NULL,
       path = NULL, scale = 0.65, width = NA, height = NA,
       units = "in", dpi = 300)



#-------------EXPORTING DATA TO CSV--------------
#-----------------------------------------------

island.mean.summary = fox.adult %>%
  group_by(Island) %>%
  summarise_at(c("ECV", "BM", "EQ"), mean)

subspecies.mean.summary = fox.adult %>%
  group_by(Subspecies) %>%
  summarise_at(c("ECV", "BM", "EQ"), mean)

island.median.summary = fox.adult %>%
  group_by(Island) %>%
  summarise_at(c("ECV", "BM", "EQ"), median)

subspecies.median.summary = fox.adult %>%
  group_by(Subspecies) %>%
  summarise_at(c("ECV", "BM", "EQ"), median)

#Island means and medians
write.csv(island.mean.summary,"Island Mean Summary.csv", row.names = FALSE)
write.csv(island.median.summary,"Island Median Summary.csv", row.names = FALSE)

#Subspecies means and medians
write.csv(subspecies.mean.summary,"Ssp Mean Summary.csv", row.names = FALSE)
write.csv(subspecies.median.summary,"Ssp Median Summary.csv", row.names = FALSE)
```

```
#Regression statistical tests
write.csv(reg.summary,"Regresssion Stat Summary.csv", row.names = TRUE)
write.csv(reg.summary2,"Regresssion Stat Summary TSL.csv", row.names = TRUE)
write.csv(an.mod.sp,"Regresssion ANOVA Stat Summary.csv", row.names = TRUE)



#-------------MAPS OF SPECIMEN LOCATIONS---------------
#-------------------------------------------------------

#--Setting up dataframe of specimen locations--
#Removing unknowns
g.coord2 = data.frame(na.omit(g.coord))

#Converting coordinates to sf system
point.sf = st_as_sf(g.coord2, coords = c("Longitude", "Latitude"), crs = 4326)

#Get all states/provinces globally
geo.reg = ne_states(country = c("Canada", "United States of America", "Mexico"),
                    returnclass = "sf")
#Name key regions where foxes are located
main.regions = c("California", "Arizona", "New Mexico",
                  "Sonora", "Jalisco", "Colima")

#Assign colors to featured regions
geo.reg = geo.reg %>%
  mutate(highlight = ifelse(name %in% main.regions, "Highlighted", "Other"))

#Importing Continental Divide Data
divide = st_read("/Continental_Divide-Pacific_Atlantic/Continental_Divide-
Pacific_Atlantic.shp")

#Plot with highlighted states
ggplot(data = geo.reg) +
  #Adding map of North America
  geom_sf(aes(fill = highlight), color = "slategray", size = 0.3) +
  scale_fill_manual(values = c("Highlighted" = "white", "Other" = "gray95")) +
  #Adding specimen data points
  geom_sf(data = point.sf, aes(color = Subspecies, shape = Detail), size = 5) +
  #Adding Ellipses around Groups
  stat_ellipse(data = g.coord2, na.rm = TRUE,
               aes(x = Longitude, y = Latitude, color = Subspecies),
               type = "norm", level = 0.95, size = 1) +
  scale_color_manual(values = subspec.col[7:10]) +
  scale_shape_manual(values = c(13, 18)) +
  #Continental Divide Line
  geom_sf(data = divide, color = "blue", size = 1, linetype = "dashed") +
  #Setting Map Boundaries
  coord_sf(xlim = c(-125, -95), ylim = c(15, 45), expand = FALSE) +
  #Adding Scale Bar
  annotation_scale(location = "bl", width_hint = 0.25, style = "ticks") +
  theme_minimal() +
  labs(title = "Gray fox Specimen Locations",
       fill = "")

ggsave("Map_Gray Fox_Specimens.pdf", plot = last_plot(), device = NULL,
       path = NULL, scale = 0.8, width = NA, height = NA,
       units = "in", dpi = 300)


#--Generating general map of the Channel Islands--

#Importing coastline data
coast1 = st_read('/N30W120/N30W120.shp')
coast2 = st_read('/N30W125/N30W125.shp')
```

```
#Combining dataset
coastfull = rbind(coast1, coast2)

#Get state/province-level data for US and Mexico
states = ne_states(country = c("United States of America", "Mexico"),
                   returnclass = "sf")

# Plot Islands
ggplot(data = states) +
  geom_sf(fill = "white", color = NA, size = 0.3) +
  #Coastline
  geom_sf(data = coastfull, color = "slategray", size = 0.3) +
  coord_sf(xlim = c(-121, -117), ylim = c(32.5, 34.5),
    expand = FALSE) +
  #Adding Scale Bar
  annotation_scale(location = "bl", width_hint = 0.25, style = "ticks") +
  theme_minimal() +
  labs(title = "Channel Islands of California", fill = "")

ggsave("Map_Channel Islands.pdf", plot = last_plot(), device = NULL,
       path = NULL, scale = 0.8, width = NA, height = NA,
       units = "in", dpi = 300)


# --- Bathymetry Map of SantaRosae ---

#Pulling public bathymetry data from NOAA for desired region
channel.isl = getNOAA.bathy(lon1 = -121, lon2 =-118,
                            lat1 = 33.5, lat2 = 34.5, resolution = 0.1)

#Generating rough plot
blues = colorRampPalette(c("red","purple","blue", "cadetblue1","white"))
plot(channel.isl, image = TRUE, bpal = blues(100))


#Generating info for GGplot
chan = as.xyz(channel.isl)

ggplot() +
  #Using bathymetry depth
  geom_tile(data = chan, aes(x = V1, y = V2, fill = V3)) +
  #Setting bath depth color pallette
  scale_fill_viridis_c() + # Example: Viridis color palette
  coord_sf(crs = st_crs(4326)) + # Use a geographic CRS (e.g., WGS 84)
  #Adding an outline of bathymetry data at -120 meters from shoreline
  geom_contour(data = chan, aes(x = V1, y = V2, z = V3),
               breaks = -120, color = "black", linewidth = 0.5) +
  #Adding outline of current shoreline and islands
  geom_sf(data = coastfull, color = "slategray", size = 0.3) +
  labs(x = "Longitude", y = "Latitude", fill = "Depth (m)") +
  theme_bw()




## BONUS ##

#Calculating body mass from Van Valk TSL
BM2.fun = function(tsl) 10^(2.86*(log10(tsl)) - 5.21) #Canidae only
BM2 = BM2.fun(raw.dat$TSL)
#Van Valk Comparison
fox.BM.comp = cbind(raw.dat, BM, BM2)
#Isolating adults for mean comparisons
```

```
fox.BM.comp = fox.BM.comp[fox.BM.comp$Age == "Adult",]

BM.comp.mean.summary = fox.BM.comp %>%
  group_by(Island) %>%
  summarise_at(c("BM", "BM2"), mean)


#Means from Van Valkenburgh not consistent with known measurements,
#proceeded with use of Engelman equation
```
