# Supplementary material for: Increased brain size of the dwarf Channel Island fox (Urocyon littoralis) challenges “Island Syndrome” and suggests little evidence of domestication
Source: PLoS One. 2025 Aug 20;20(8):e0328893. doi: 10.1371/journal.pone.0328893 (PMC12367152; doi:10.1371/journal.pone.0328893)
Supplement: Attachment — Submitted filename: Schoenberger et al_Island Fox_PLOS One_Response to Reviewers.pdf [file pone.0328893.s011.pdf]

Carlo Meloro, Academic Editor
PLOS ONE
1875 Mission Street, Suite 103 #188 San Francisco, CA 94103

May 11, 2025

Dear Carlo Meloro and reviewers,

Thank you for your comments and advice on revisions for our research article, "Increased brain size of the dwarf Channel Island fox (*Urocyon littoralis*) challenges 'Island Syndrome' and suggests little evidence of domestication". Our responses are shown *in italics* below.

Editor comments:

Intro notes:

1. "First clarify the taxonomic scale of study. If subspecies is available for grey fox this should be considered and implemented."
    a. *We have updated the manuscript to explicitly state the taxonomic scale of the study, outlining the gray fox subspecies used and the geographic distribution of mainland specimens (Lines 80-90, 134-135, 144-154, 271-286, Fig 1A, Fig 6, S1 Fig).*
2. "Sexual dimorphism should also be tested more explicitly together with the implementation of stats tests for slope between grey and island fox."
    a. *Sexual dimorphism has been previously examined in the island and gray fox by multiple other studies and has now been outlined explicitly in this manuscript (Lines 71-73), with additional comments about the EQ sex differences from our findings (Lines 31, 261-262, S2 Fig).*
3. "If available, report the area of each island and run a simple correlation test between average skull length and island area."
    a. *The area of each island is noted in Table 1, and body size among islands has been added from prior studies (Lines 65-68), with little correlation between body size and land area in the island fox.*
4. "Provide raw data as appendix or in a repository and implemented literature and other concepts as advised by reviewers."
    a. *Raw data of endocasts has been added to a Morphosource repository.*

Journal Requirements:

1. "Please ensure that your manuscript meets PLOS ONE's style requirements, including those for file naming."
    a. *Manuscript has been updated to match style and file naming requirements for PLOS One.*
2. "In your manuscript, please provide additional information regarding the specimens used in your study. Ensure that you have reported human remain specimen numbers and complete repository information, including museum name and geographic location."
    a. *Permits and specimen info have been added to comply with PLOS One requirements (Lines 130-132).*
3. "We note that you have provided funding information that is not currently declared in your Funding Statement. However, funding information should not appear in the Acknowledgments section or other areas of your manuscript."
    a. *No funding has been provided for this project specifically, comments regarding funding in the acknowledgements were regarding general funding and support for Kimberly's PhD. Phrasing in Acknowledgments has been changed to refer to only the support provided (Lines 480-483), and funding statement declaration has been updated.*
4. "We note that Figure 1 in your submission contain [map/satellite] images which may be copyrighted."
    a. *Figure 1 has been modified to use public datasets for the main body which are cited explicitly in the figure caption, and copyright permission has been obtained from Cambridge University Press, with proof of permission uploaded along with this revised submission.*

5. “Please review your reference list to ensure that it is complete and correct.”
    a. *Reference list has been updated to accommodate requested revisions, with some changes to the order of the original references as edits were made. The following references added:*
        i. *Meiri S, Simberloff D, Dayan T. Insular carnivore biogeography: Island area and mammalian optimal body size. American Naturalist. 2005;165(4):505–14. doi: 10.1086/428297*
        ii. *Schutz H, Polly PD, Krieger JD, Guralnick RP. Differential sexual dimorphism: size and shape in the cranium and pelvis of grey foxes (Urocyon). Biological Journal of the Linnean Society. 2009;96(2):339–53. doi: 10.1111/j.1095-8312.2008.01132.x*
        iii. *Raia P, Barbera C, Conte M. The fast life of a dwarfed giant. Evol Ecol. 2003;17(3):293–312. doi: 10.1023/A:1025577414005*
        iv. *Reding DM, Castañeda-Rico S, Shirazi S, Hofman CA, Cancellare IA, Lance SL, et al. Mitochondrial Genomes of the United States Distribution of Gray Fox (Urocyon cinereoargenteus) Reveal a Major Phylogeographic Break at the Great Plains Suture Zone. Front Ecol Evol. 2021;9. doi: 10.3389/fevo.2021.666800*
        v. *Hofman CA, Rick TC, Hawkins MTR, Funk WC, Ralls K, Boser CL, et al. Mitochondrial Genomes Suggest Rapid Evolution of Dwarf California Channel Islands Foxes (Urocyon littoralis). PLoS One. 2015;10(2):e0118240. doi: 10.1371/journal.pone.0118240*
        vi. *Gompper ME, Petrites AE, Lyman RL. Cozumel Island fox (Urocyon sp.) dwarfism and possible divergence history based on subfossil bones. J Zool. 2006;270(1):72–7. doi: 10.1111/j.1469-7998.2006.00119.x*
        vii. *Collins PW. Origin and Differentiation of the Island Fox: A Study of Evolution in Insular Populations [Master of Arts]. Santa Barbara: University of California; 1982.*
        viii. *Massicotte P, South A. rnaturalearth: World Map Data from Natural Earth. CRAN: Contributed Packages. 2017. doi: 10.32614/CRAN.package.rnaturalearth*
        ix. *National Geodetic Survey. NOAA National Shoreline Data Explorer [Shapefile]. https://nsde.ngs.noaa.gov/. National Oceanic and Atmospheric Administration; [accessed 30 Apr 2025] Available from: https://nsde.ngs.noaa.gov/*
        x. *McGee S. Continental Divide-Pacific/Atlantic [Shapefile]. ArcGIS Hub. U.S. Fish & Wildlife Service; 2023. [accessed 29 Apr 2025] Available from: https://hub.arcgis.com/datasets/fws::continental-divide-pacific-atlantic/about*
        xi. *Rozzi R. Palaeobiogeography and evolution of insular bovids: ecogeographic patterns of body mass variation and morphological changes (Unpublished Ph.D Thesis). Sapinza Università di Roma; 2013.*
        xii. *Liakopoulou D, Roussiakis S, Lyras G. The brain of Myotragus balearicus , an insular bovid from the Balearics. Hist Biol. 2024;1–8. doi: 10.1080/08912963.2024.2406962*
        xiii. *Lyras G. The evolution of the brain in Canidae (Mammalia: Carnivora). Scr Geol. 2009;139.*
        xiv. *Floyd CH, Van Vuren DH, Crooks KR, Jones KL, Garcelon DK, Belfiore NM, et al. Genetic differentiation of island spotted skunks, Spilogale gracilis amphiala. J Mammal. 2011;92(1):148–58. doi: 10.1644/09-MAMM-A-204.1*
        xv. *Bolas EC, Quinn CB, Van Vuren DH, Lee A, Vanderzwan SL, Floyd CH, et al. Pattern and timing of mitochondrial divergence of island spotted skunks on the California Channel Islands. J Mammal. 2022;103(2):231–42. doi: 10.1093/jmammal/gyac008*
        xvi. *Pasciullo Boychuck S, Brenner LJ, Gagorik CN, Schamel JT, Baker S, Tran E, et al. The gut microbiomes of Channel Island foxes and island spotted skunks exhibit fine-scale differentiation across host species and island populations. Ecol Evol. 2024;14(2). doi: 10.1002/ece3.11017*

Additional Editor Comments:

General:

1. “The paper reads well and it is nice however my main point of concern is your way of considering U. cinereoargenteus as a single species vs 6 subspecies. In theory, your paper will be much stronger if you could account for subspecies differences also within your grey fox sample. Also you should introduce sexual dimorphism earlier in the paper and test for it.”
    a. *As mentioned above, subspecies differences within the gray fox sample have been explicitly addressed and tested throughout the manuscript (see line numbers above). Sexual size dimorphism has also been addressed in the paper, including reference to the suggested Raia et al. (2003) paper (Lines 71-75). As SSD has already been largely previously examined in other papers, I refer to those studies and thus did not compute other SSD ratios explicitly with my dataset besides those regarding EQ (S2 Fig).*
2. “I think the paper include quite valuable data and information. If you could make raw data available [eg. measurements at least] that will also be good.”
    a. *Raw data is available in the supplement as S1 Table containing all linear and volumetric measurements for specimens, with collection information and catalog numbers. I have updated the supplement title to explicitly state that it contains the measurement info along with the specimen list.*

Line comments:

Introduction

1. “Line 58-59: add scientific names since this is first time you mention these species”
    a. *Agreed, corrected (Lines 62-64).*
2. “Line 66: perhaps refer to Figure 1 and add some arrows on the map showing the potential route of island colonization with dates. Add in the map also a mini-map outlining the area within the broader context of North American continent”
    a. *Agreed, updated with above comments as well as including a map of the gray fox specimen areas in the context of North America (Fig 1).*

Material and Methods

3. “Line 92: since you refer to the island subspecies it is worth providing more details on your sample distribution for U. cinereoargenteus for which there have been recorded about 16 subspecies....”
    a. *Details for gray fox specimens have been updated here and throughout the paper (see above comments).*
4. “Line 126: do you mean cranial morphometry? You do not present any geometric morphometric analysis that is also based upon anatomical landmarks but in geometric morphometrics the raw data are coordinates and NOT linear distances between landmarks.”
    a. *Yes, corrected (Line 183).*
5. “Line 138: nice one....I wonder how does this compare to the Van Valkenburgh equations on Canidae only. It would make more sense to use Canidae specific equation for a more accurate body mass reconstruction”
    a. *Equations from below reference (Van Valkenburgh, 1990) use four measurements (head-body length, skull length, occiput-orbit length, and lower first-molar length). The only measurement that we were able to test for comparison without collection additional measurements from all specimens was the skull length, which we tested with Van Valkenburgh’s equation to calculate body mass. This resulted in substantial overestimates of known fox body mass. Mean body mass for all islands using Van Valkenburgh equations was well above 3 kg, which is higher than known body mass ranges for island foxes (1.2-2.7 kg). To contrast, averages calculated using our original method of Engelman’s regression of the occipital condylar width (OCW) fell within the known range (Island means ranged between 1.98-2.64 kg). As such, we did not proceed with using the suggested equation.*
        i. *Van Valkenburgh, B. 1990. Skeletal and dental predictors of body mass in carnivores. In: J. Damuth and B.J. MacFadden (eds.), Body Size in Mammalian Paleobiology, 1–11. Cambridge University Press, Cambridge.*

Results

6. "Line 161: because you are talking about scaling I was expecting a test of slope differences and not a test of variance. It might be worth exploring the regression of Skull length (X) vs Brain Size between the two subspecies or different subspecies. This test will address the question: does grey fox and island fox brain size scale in the same way? Also I can see Sex in Figure S1 -this should be in the paper since it is your very first result- but Sex is not mentioned in the Introduction / Mat-Methods...if you got sexual dimorphism you should first test for differences in relative brain size between species and sex…"
    a. *Agreed, added info for testing slope differences in both the original plot (Body Mass vs Brain Size) and new additional plot (Skull Length vs Brain Size). Info can be found in Fig 4 and Table 2.*
    b. *Figure S1 has been moved to the main manuscript as Fig 3. Sexual dimorphism has been addressed in the intro (Lines 71-73) and in S2 Fig.*
7. "Line 162: why did you use Wilcoxon [generally it applies to dependent data that have a time structure [e.g. before and after]...you should use simple t-test or non-parametric Mann-Whitney if your groups are species."
    a. *This has been corrected, using non-parametric Mann-Whitney (Line 230).*
8. "Line 165: Report slopes and intercepts (and 95% CI if necessary) for your models in a table....slopes of the two Urocyon seem parallel to me but without any stats test we will never know that for sure...try and test for difference using ANCOVA and eventually check if slope differ between subspecies (if n within subspecies is big enough)."
    a. *Slopes and intercepts of each species with tests for significant differences have been added in Table 2.*
9. "Line 177: delete "with""
    a. *Agreed, deleted.*
10. "Line 179-181: leave it out for the discussion"
    a. *Agreed, removed.*
11. "Line 191: you cannot talk of shape for plot 5B -it is mainly size, right?-. Different thing is plot 5C. A log transformation of the data might make the plot also a bit better. You can clearly see the issue of species (big yellow convex hull) vs subspecies (convex hulls there are much much smaller)"
    a. *Agreed, results have been updated to clarify that differences in raw data are driven by size (Lines 281-286). PCA has been rerun with gray fox subspecies included (Fig 6).*
12. "Line 192-197: you should test for differences using MANOVA/CVA...if test is significant then with post-hoc you can identify the pair of taxa that are more disparate between each other...based on the plot there seems to be no difference between subspecies of island fox so statistically it is more correct to not to consider the populations as separate analytical entities…"
    a. *Agreed, information about additional statistical tests has been added to the manuscript under the methods and results sections (Lines 220-225, 268-281).*
13. "Line 225: if you manage to find subspecies for U. cinereoargenteus perhaps your differences might be even larger...at the moment the only true difference I feel to trust if the one between grey and island fox."
    a. *Significance tests were run for species-species comparisons as well as subspecies-subspecies comparisons. Species showed substantial significant differences, which was consistent with the subspecies-subspecies pairings and noted in the results section (Lines 268-281).*

Discussion

14. "Line 269: nice explanation perhaps it is worth talking also about the competitors...do they have many on the island or reduced competition (check for theoretical background: Raia, P., & Meiri, S. (2006). The island rule in large mammals: paleontology meets ecology. Evolution, 60(8), 1731-1742)? does diet change between gray and island fox? any refs on diet?"
    a. *Agreed, info about competitors (only one is known (island spotted skunk) and only on two of the islands) has been added into the discussion section (Lines 384-394).*
15. "Line 290: you can check for this in your data testing the association between average skull size and island area for the six island subspecies (van der Geer, A. A., van den Bergh, G. D., Lyras, G. A., Prasetyo, U. W., Due, R. A., Setiyabudi, E., & Drinia, H. (2016). The effect of area and isolation on insular dwarf proboscideans. Journal of Biogeography, 43(8), 1656-1666)"

  a. *This info has been added in the introduction (Lines 65-68), citing other studies that already show lack of correlation between island fox subspecies body size and island area.*

Reviewer comments:

Reviewer #1:

1. "One of the most significant aspects of this paper is the size of its sample. Most studies on the brain size of insular mammals rely on fossil specimens, often with very limited sample sizes (sometimes just a single specimen). In contrast, this paper examines 297 specimens, which is a considerable strength that could be emphasized more."
   a. *Agreed, added to the materials section (Lines 137-139).*
2. "The authors noticed that the island fox has a reduced length in the prefrontal area. I believe this is a consequence of its shorter rostrum. In general, canids that have relatively short faces have relatively high and massive frontal brain lobes. A somewhat similar case to Urocyon is the living raccoon dog Nyctereutes procyonoides. That species has a shorter muzzle than its Pliocene relatives. The muzzle shortening led to a shortening of the proreal gyrus length (Lyras 2009)."
   a. *Agreed, added to the discussion section (Lines 381-383).*
3. "On Table 2, I have three minor comments: 1. Csiki-Sava et al. (2018) compare Homo floresienceis with an early form of Homo erectus 'Homo erectus (early form)'. I suggest they add a similar parenthesis in their table, as brain sizes differ significantly between early and late forms of Homo erectus. 2. They list Gallogoral meneghinii as the ancestor of Myotragus balearicus. Actually that is not true, and neither the reference they cite (Köhler et al., 2004), says so. Gallogoral meneghinii is a Pleistocene relative of Myotragus. 3. They list Cervus elaphus as the ancestor of Candiacervus ropalophorus. It has been suggested that the fallow deer is the living relative of Candiacervus (van der Geer, 2018). I am sure that the replacement of Cervus elaphus with Dama dama will not significantly alter the values in the table."
   a. *1. Agreed, updated table info for Homo erectus.*
   2. Agreed, and this was the reason for using Gallogoral in the first place. Info on actual relationships has been added to the table caption.
   3. Good catch - originally this manuscript used values from Cervus elephas then updated to using values from Dama dama in calculated changes, but had mistakenly not updated the species name in the table. This has been corrected with the accurate comparison species (Dama dama), with EQ and body mass change values remaining the same. Authors have further verified that all values and species are accurate in revised manuscript.

Reviewer #2:

1. "I selected no for data availability, because this is not clear to me: are the CT-scans available for external brain morphology?"
   a. *The CT scans have been uploaded to be publicly available on Morphosource, with the link added under "Data Availability" (Lines 486-488).*
2. "Then, in the Introduction, line 51, what means 'simplified locomotion'? I'm not aware of such a feature in island endemic mammals, but my guess is that here reduced dispersal abilities are meant, such as loss of running in vertebrates, of flight in birds. In any case, 'simplified' needs to be explained here. Same with coloration, it's rather dull than simplified, and applies only (?) birds (mammals can e.g. develop spotted patterns, white-tipped tail etc., which is not 'simplified coloration'."
   a. *Those were the intended ideas of the original phrasing, but agreed that it needs to be more group-specific. Phrasing has been updated for clarity (Line 54).*
3. "Further, in line 60 no reference is given for the body size reduction in the island foxes (could be Lyras et al. 2010 JoB; but perhaps there is a more recent reference)."
   a. *Agreed, citation added (Line 64).*
4. "Then, page 4 lines 82-83 the definition of endemic includes also mainlands, so the addition 'island' or 'insular' is needed here. There are many more living endemic canids, but if you excluse the mainlands, then the statement is almost true (there is also the Cozumel island fox)."

   a. *Agreed, this was the intent of the original statement, which referred to species ONLY living on islands (rather than both on islands and the mainland). Phrasing has been updated to clarify (Lines 68-71). Info on Cozumel fox has also been added to introduction (Lines 91-97).*

5. "Discussion page 13 line 239 reduced predatory pressure does not apply mostly to small vertebrates; I'd argue the opposite. Elephants evolve dwarfism when no predators are around, and they are not small. Also, for rodents, predatory pressure is not lower on islands, but shifted to birds only (which can grow gigantic)."
   a. *Agreed, phrasing has been updated to clarify patterns more explicitly (Lines 330-332).*
6. "Page 15 last paragraph, this link with increased arboreality is very interesting, and looks like a strong point. So I was wondering, how is the situation on San Nicolas?"
   a. *San Nicolas does not possess any trees, which would make sense with the lower EQ values. This info has been added to the discussion section regarding San Nicolas (Lines 403-405).*
7. "Page 16 line 316 I don't agree that the first domestic dogs in the late Pleistocene were mostly hunting assistants; archaeological evidence suggests rather that they were primarily food items (as with horses)."
   a. *This phrasing was intended to indicate that the early dogs were not specifically used for companionship, but rather only utilitarian reasons. Phrasing has been updated to include use as food items (Line 425).*
8. "Lastly, in the bibliography, a few titles are erroneously given in caps"
   a. *Thank you, this has been corrected.*

Changes as noted above are reflected in the revised manuscript. Thank you again for your time and we look forward to hearing from you.

Sincerely,

Kimberly A. Schoenberger
PhD Candidate, University of Southern California
Graduate Student in Residence, Natural History Museum of Los Angeles County
kschoenb@usc.edu
