# Supplementary material for: Increased brain size of the dwarf Channel Island fox (Urocyon littoralis) challenges “Island Syndrome” and suggests little evidence of domestication
Source: PLoS One. 2025 Aug 20;20(8):e0328893. doi: 10.1371/journal.pone.0328893 (PMC12367152; doi:10.1371/journal.pone.0328893)
Supplement: Attachment — Submitted filename: Urocyon_Tests.pdf [file pone.0328893.s012.pdf]

PCA -using the Var-Covariance matrix is better because all your measurements are on the same scale. You will note that the level of overlap for littoralis subspecies on the left is similar to that of cinereoargenetus subspecies on the right. Note also that using Var-Cov matrix shows you that PC1 now explains much more (84%). We can use for pairwise tests and MANOVA only the first five PCs that collectively explains almost 96% of var.

PC	Eigenvalue	% variance
1	0.0110884	84.227
2	0.000687191	5.2199
3	0.000388053	2.9476
4	0.000246699	1.8739
5	0.000223446	1.6973
6	0.000144714	1.0992
7	0.000123472	0.93789
8	9.10E-05	0.69103
9	6.20E-05	0.47095
10	5.14E-05	0.39031
11	3.43E-05	0.26079
12	2.43E-05	0.1843

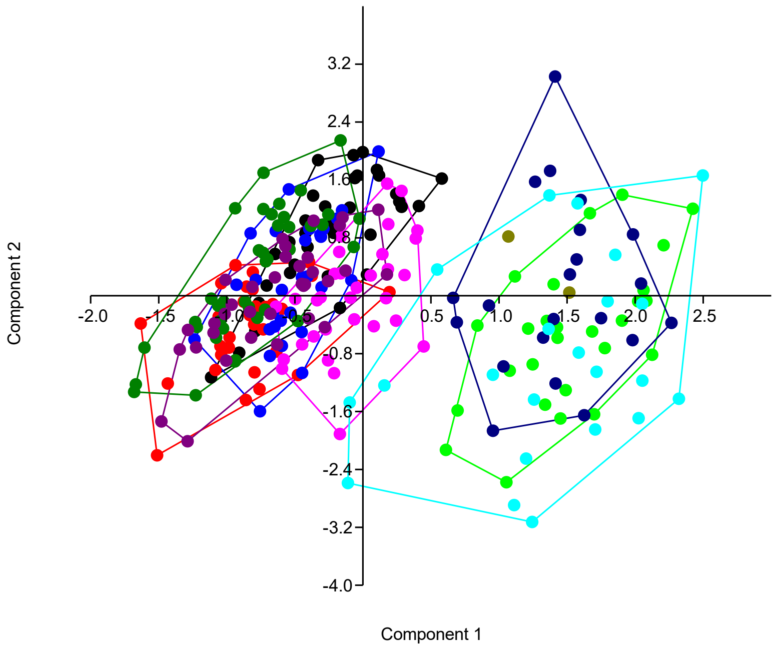

Loadings of component 1 show a positive correlation between PC1 and all the variables (the size axis), on PC2 you got positive association especially on BCVV

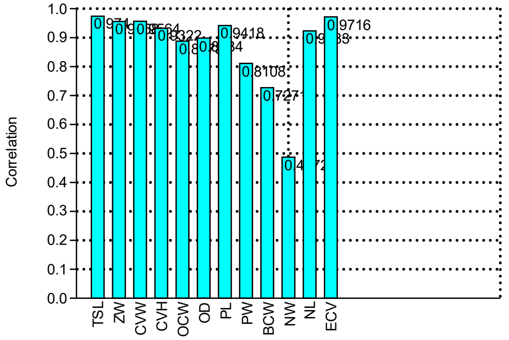

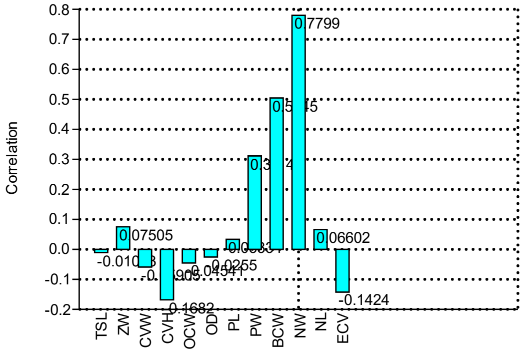

Doing MANOVA/CVA using PAST also using subspecies as coherent groups give a Wilk's lambda = 0.04234, df = 45, 1148, F = 26.22, $p < 0.0001$.

On the table below there is Hotelling's pairwise difference (below is not corrected by multiple comparison, pay attention only to the value above in bold, they are Bonferroni corrected). You might note that *U.c.californicus* differed significantly from *U.c.nigrirostris*.

The same test using only PC1 and PC2 show no difference between *californicus* and *nigrirostris*. By doing an ANOVA on every single cranial variable I noted significance only for NW to test for differences between U. cinereoargenteus subspecies (see table in the next page) so you are probably good to go by merging all in one taxon as you did in the paper.

	littoralis	santacruzae	santarosae	catalinae	clementae	dickeyi	californicus	nigrirostris	scottii	townsendi
littoralis	0	**2.95E-07**	**0.0458853**	**0.000456034**	**0.000719629**	**9.94E-10**	**2.51E-25**	**2.79E-20**	**2.13E-22**	**0.00120935**
santacruzae	6.56E-09	0	**0.0349818**	**5.56E-08**	**0.0181148**	**4.33E-12**	**5.28E-27**	**3.67E-22**	**1.83E-23**	**6.99E-05**
santarosae	0.00101967	0.000777373	0	0	**1.94E-10**	**6.95E-10**	**4.25E-21**	**6.13E-16**	**5.20E-18**	**0.0454474**
catalinae	1.01E-05	1.24E-09	0.000191299	1.94E-10	0	**1.62E-12**	**4.33E-32**	**1.05E-26**	**1.82E-28**	**4.75E-06**
clementae	1.60E-05	0.00040255	0.000139989	4.32E-12	0	**3.60E-14**	**9.62E-34**	**2.33E-28**	**4.05E-30**	**1.05E-07**
dickeyi	2.21E-11	9.62E-14	2.31E-08	1.54E-11	3.60E-14	0	**1.15E-27**	**6.15E-22**	**1.08E-23**	**0.000314394**
californicus	5.57E-27	1.17E-28	3.60E-26	9.45E-23	9.62E-34	2.56E-29	0	**0.00104111**	1	1
nigrirostris	6.20E-22	8.15E-24	6.52E-21	1.36E-17	2.33E-28	1.37E-23	2.31E-05	0	0.0501737	1
Scottii	4.73E-24	4.07E-25	8.32E-23	1.16E-19	4.05E-30	2.41E-25	0.0887941	0.00111497	0	1
townsendi	2.69E-05	1.55E-06	3.32E-05	0.00100994	1.05E-07	6.99E-06	0.089757	0.950104	0.183673	0

ANOVA using a sample of 71 U. cinereoargenteus specimens testing for subspecies differecne

		Sum of Squares	df	Mean Square	F	Sig.
TSL	Between Groups	63.432	3	21.144	.766	.517
	Within Groups	1877.269	68	27.607		
	Total	1940.702	71			
ZW	Between Groups	31.391	3	10.464	1.664	.183
	Within Groups	427.634	68	6.289		
	Total	459.025	71			
CVW	Between Groups	3.983	3	1.328	.625	.601
	Within Groups	144.364	68	2.123		
	Total	148.347	71			
CVH	Between Groups	8.053	3	2.684	1.886	.140
	Within Groups	96.761	68	1.423		
	Total	104.814	71			
OCW	Between Groups	3.945	3	1.315	1.233	.304
	Within Groups	72.514	68	1.066		
	Total	76.459	71			
OD	Between Groups	2.728	3	.909	2.088	.110
	Within Groups	29.609	68	.435		
	Total	32.336	71			
PL	Between Groups	33.337	3	11.112	.935	.429
	Within Groups	808.593	68	11.891		
	Total	841.930	71			
PW	Between Groups	7.616	3	2.539	1.624	.192
	Within Groups	106.332	68	1.564		
	Total	113.948	71			
BCW	Between Groups	2.106	3	.702	.894	.449
	Within Groups	53.400	68	.785		
	Total	55.506	71			
NW	Between Groups	5.201	3	1.734	3.847	.013
	Within Groups	30.646	68	.451		

	Total	35.847	71			
NL	Between Groups	18.001	3	6.000	.881	.455
	Within Groups	463.085	68	6.810		
	Total	481.085	71			
ECV	Between Groups	5.949	3	1.983	.174	.913
	Within Groups	773.689	68	11.378		
	Total	779.638	71			
