# Supplementary material for: Increased brain size of the dwarf Channel Island fox (Urocyon littoralis) challenges “Island Syndrome” and suggests little evidence of domestication
Source: PLoS One. 2025 Aug 20;20(8):e0328893. doi: 10.1371/journal.pone.0328893 (PMC12367152; doi:10.1371/journal.pone.0328893)
Supplement: Attachment — Submitted filename: Schoenberger et al_Response to Reviewers-2.1.docx [file pone.0328893.s014.docx]

Carlo Meloro, Academic Editor

*PLOS ONE*

1875 Mission Street, Suite 103 #188 San Francisco, CA 94103

July 3, 2025

Dear Carlo Meloro and reviewers,

Thank you again for your comments and advice on revisions for our research article, “Increased brain size of the dwarf Channel Island fox (*Urocyon littoralis*) challenges ‘Island Syndrome’ and suggests little evidence of domestication”. Specific line responses are shown *in italics* below. Note: line numbers below refer to corrections shown in the clean manuscript file. Content is the same in both the clean and markup files, but Microsoft Word has known inconsistencies in line numbering while using “track changes”, so there are some discrepancies in the line numbering between the files despite the writeups being identical.

**Editor comments:**

Thank you for providing examples of the plot types and calculations you were looking for in this revision. I would like to note that in the example you provided, it appears you also included the “ECV” in the calculations for the PCA and loading values. I have updated the statistical tests using PAST and your other recommendations, but please note that the values will be slightly different from the ones in your example as they do not include ECV due to it being a volumetric measure. They will also differ due to removal of an outlier and clustering of gray fox into one group.

Additionally, copyrighted content has been removed from Figure 1 and replaced with a map generated using only datasets compliant with CC BY 4.0 license. Figure caption has been updated (Lines 161-162).

**Line comments:**

1. Line 193: "Carnivora" upper case
   a. *Corrected.*
2. Line 210: It is ANCOVA and NOT ANOVA. Modify as: "We then used ANCOVA, including interaction terms, to test whether slopes and intercepts of these scaling relationships differed significantly between species".
   a. *Corrected (Lines 212-213).*
3. Line 216: I do not think you used "Welch's ANOVA". This is used when variances are not equal, in that case the post hoc should be Dunnett's T3.
   a. *Welch’s ANOVA was initially used due to unequal variance, but revisions have been made per suggestions. Statistical tests used Levene’s test to test for equal variance. Variance was not equal, so proceeded to use one-way ANOVA to assess for statistical differences among group means. Post hoc tests implemented Dunnett’s T3 as suggested. (Lines 214-221).*
4. Line 223-224: it is ok to perform MANOVA only on PC1 and PC2 although you might try to cover at least 95% of total variance (e.g. first 5 PCs) and provide results as expressed by Wilk's lambda and Pillai Trace. Your PCA was probably based on Correlation matrix...you should use Covariance matrix if your input variables are log transformed measurements. See the attach doc "Urocyon_tests". I selected only Adult specimens and provided some examples on analyses run using PAST and SPSS. It is fine to combine all U. cinereoargenteus subspecies but still you need to implement MANOVA more appropriately. ANOVA that follows MANOVA is not appropriate, so please use PAST or try to use simple script in R to run Hotelling's pairwise test after MANOVA.
   a. *Thank you for providing an example of statistical tests! We ended up using PAST for the revised PCA using the variance-covariance matrix, clustering the gray fox as one group. Description of methods outlined in Lines 222-231, and results in Lines 273-289. Ease of use in PAST allowed us to use all principal components for MANOVA and post hoc tests. Statistical differences shown in newly added Table 3 in the manuscript (Line 299). PCA plots generated in R were updated to use the variance-covariance matrix and were checked against plots generated in PAST to ensure consistency. Use of R for plots was chosen to keep figure designs consistent throughout the paper. Figure 6 has been updated to reflect the above changes (Line 291).*
5. Report pairwise differences after MANOVA not based on each single PC.
   a. *Addressed above, see new Table 3 (Line 299).*
6. Please clarify if you removed the Juveniles from the sample, I think they should be removed.
   a. *Juveniles were not included in the sample, added note to state this explicitly (Lines 223-224).*
7. Figure 4: It is not clear to what line the R2 and p value refers to. Report eventually the general equation and the R2 next to the general regression line it refers to or use a legend.
   a. *Agreed, Figure 4 has been updated to include a clearer legend and addition of general equations for each regression. Figure caption updates are on Lines 250-254,*
8. Figure 5 A and B are identical...perhaps put one or the other in the Appendix
   a. *Figure 5A has been left in the main text (Lines 260-262, 269), 5B moved to the appendix S2 fig (Lines 262-264).*
9. Figure 6: you got one outlier for Fig 6A and 6C… I noted from your table that you also measured Juveniles...they should be excluded.
   a. *Thank you, the outlier was identified and removed for PCA revisions and noted in the text (Lines 229-230). Juveniles were not included in the original PCA, but this has now been outlined explicitly in the text (Lines 223-224).*

**Additional changes to be noted:**

1. Figures and supplement:
   a. Figs 2-3, Fig 7, S1 Fig, S1 Table, and S2 Table are the same as previous submission with no changes.
   b. Fig 1 has been updated to replace copyrighted content with CC BY compliant data.
   c. Fig 4 has been updated to include general regression equation and added clarity.
   d. Fig 5 has been updated to only include Fig 5A from previous submission, 5B moved to supplement.
   e. Fig 6 has been updated with var-covar PCA plots and clustering gray fox subspecies into one group.
   f. S1 Appendix has been updated to reflect new code.
   g. S2 Fig is the new supplementary figure, previously Fig 5B.
   h. S3 Fig is the previous S2 Fig.
2. DOIs have been generated for supplemental Morphosource files, which have now been included under the Data Availability Statement on Lines 499-501.
3. Two references were added for NOAA bathymetry data (Line 593) and PAST Software (Line 610):
   a. *NOAA National Centers for Environmental Information. ETOPO 2022 15 Arc-Second Global Relief Model. 2022. doi:10.25921/fd45-gt74*
   b. *Hammer Ø, Harper DAT, Ryan PD. PAST: Paleontological statistics software package for education and data analysis. Palaeontologia Electronica; 2001.*

Changes as noted above are reflected in the revised manuscript. Thank you again for your time and we look forward to hearing from you.

Sincerely,

Kimberly A. Schoenberger

PhD Candidate, University of Southern California

Graduate Student in Residence, Natural History Museum of Los Angeles County

kschoenb@usc.edu
